# Horizontal Transfer of Microbial Toxin Genes to Gall Midge Genomes

**DOI:** 10.1093/gbe/evab202

**Published:** 2021-08-27

**Authors:** Kirsten I Verster, Rebecca L Tarnopol, Saron M Akalu, Noah K Whiteman

**Affiliations:** 1 Department of Integrative Biology, University of California, Berkeley, California, USA; 2 Department of Plant & Microbial Biology, University of California, Berkeley, California, USA; 3 Department of Molecular and Cell Biology, University of California, Berkeley, California, USA

**Keywords:** horizontal gene transfer, Diptera, toxins, cdtB, shiga toxin, lysozyme

## Abstract

A growing body of evidence has underscored the role of horizontal gene transfer (HGT) in animal evolution. Previously, we discovered the horizontal transfer of the gene encoding the eukaryotic genotoxin *cytolethal distending toxin B* (*cdtB*) from the pea aphid *Acyrthosiphon pisum* secondary endosymbiont (APSE) phages to drosophilid and aphid nuclear genomes. Here, we report *cdtB* in the nuclear genome of the gall-forming “swede midge” *Contarinia nasturtii* (Diptera: Cecidomyiidae) via HGT. We searched all available gall midge genome sequences for evidence of APSE-to-insect HGT events and found five toxin genes (*aip56*, *cdtB*, *lysozyme*, *rhs*, and *sltxB*) transferred horizontally to cecidomyiid nuclear genomes. Surprisingly, phylogenetic analyses of HGT candidates indicated APSE phages were often not the ancestral donor lineage of the toxin gene to cecidomyiids. We used a phylogenetic signal statistic to test a transfer-by-proximity hypothesis for animal HGT, which suggested that microbe-to-insect HGT was more likely between taxa that share environments than those from different environments. Many of the toxins we found in midge genomes target eukaryotic cells, and catalytic residues important for toxin function are conserved in insect copies. This class of horizontally transferred, eukaryotic cell-targeting genes is potentially important in insect adaptation.

**Significance**

The diversity of genes encoded by phages infecting bacterial symbionts of eukaryotes represents an enormous, relatively unexplored pool of new eukaryotic genes through horizontal gene transfer (HGT). In this study, we report HGT of toxin genes encoded by diverse microbial taxa to the nuclear genomes of gall midges. We found five toxin genes (*aip56*, *cdtB*, *lysozyme*, *rhs*, and *sltxB*) were transferred horizontally from bacteria, viruses, or fungi into genomes of several cecidomyiid species. Most of the toxins encoded by these genes antagonize eukaryotic cells, and we posit that they may play a role in the insect immune system.

## Introduction

There is growing evidence that horizontal gene transfer (HGT) has played an important role in animal evolution (Boto 2014; Husnik and McCutcheon 2018). HGT facilitates the evolution of novelty in animal immune systems, particularly among arthropods. Antibacterial toxins transferred from bacteria have been described in *Ixodes* ticks and in several species of Coccinellinae ladybird beetles (Hayes et al. 2020; Li et al. 2021). Some horizontally transferred genes (HTGs) have been co-opted as effectors of the insect immune system. For example, an HTG from a symbiotic virus of a parasitoid wasp, *Sl gasmin*, plays a central role in mediating phagocytosis in hemocytes of the moth *Spodoptera littoralis* (Di Lelio et al. 2019). While the role of HGT in mediating immunity against prokaryotic pathogens is well-documented, there are few characterized HGT events encoding genes that can protect animals against eukaryotic pathogens and predators (Gasmi et al. 2021).

We previously discovered HGT of a eukaryote-targeting toxin gene, *cytolethal distending toxin B* (*cdtB*), into the nuclear genomes of four insect lineages within two orders, Diptera and Hemiptera (Verster et al. 2019). *cdtB* is widespread among Proteobacteria and Actinobacteria and encodes a DNAse I-type nuclease that causes cell cycle arrest and apoptosis in eukaryotic cells (Jinadasa et al. 2011; Verster et al. 2019). The closest relatives of these insect *cdtB* copies were copies isolated from the pea aphid *Acyrthosiphon pisum* secondary endosymbiont (APSE) phages or prophages (Verster et al. 2019), which infect the secondary bacterial endosymbiont *Hamiltonella defensa* of hemipterans and other cosmopolitan symbionts like *Arsenophonus* spp. (Degnan and Moran 2008; Oliver et al. 2009, 2010; Duron 2014). APSE phages encode diverse toxins within a highly variable “toxin cassette” region of their genomes (Rouïl et al. 2020). We found another APSE toxin gene, *apoptosis inducing protein 56* (*aip56)*, fused to a paralogous copy of *cdtB* in *Drosophila ananassae* subgroup genomes (Verster et al. 2019). Since *aip56* and *cdtB* genes are proximal in APSE genomes, this further supports the HGT of toxin genes from APSE phages to insects.

Here, we report the serendipitous discovery of a full-length *cdtB* sequence in the genome of the gall midge *Contarinia nasturtii* (Diptera: Cecidomyiidae) (supplementary table S1, Supplementary Material online) (Mori et al. 2021), which is also called the “swede” midge. The Cecidomyiidae (Diptera: Nematocera) contains over 6,600 fly species with diverse life histories, behaviors, and host use patterns (Yukawa and Rohfritsch 2005; Dorchin et al. 2019; O’Connor et al. 2019; Mori et al. 2021). Many cecidomyiids are herbivorous and create destructive galls on crops (Hall et al. 2012). Interestingly, an APSE-3-like rearrangement hotspot (*rhs*) toxin gene was found in the genome of another cecidomyiid fly, the wheat pest *Mayetiola destructor* (Zhao et al. 2015). This observation, coupled with our finding of *cdtB* in *Co. nasturtii*, suggests that APSE phages may serve as a reservoir for HGT in the Cecidomyiidae.

To search for additional APSE-to-cecidomyiid HGT events (more specifically, HGT from APSE ancestors to cecidomyiid ancestors), we conducted TBLASTN searches using proteins encoded by APSE genomes as queries against all publicly available cecidomyiid whole genome sequences: *Co. nasturtii*, *M. destructor*, *Sitodiplosis mosellana, and Catotricha subobsoleta* (supplementary table S2, Supplementary Material online). We discovered several toxin-encoding genes were transferred into the genomes of the first three midge species. We used several quality control metrics to confirm these genes were integrated into the insect genome and not microbial contaminants, and then inferred the evolutionary history of these HGT events through analysis of both gene and species trees. The initial motivation of this study was to investigate the extent of HGT from APSE phage ancestors to cecidomyiids. However, we discovered that in most cases the encoded proteins were often more similar to orthologs from lineages other than APSE, such as fungi or other insect-associated viral and bacterial symbionts. Our analysis of the *δ* statistic of the phylogenetic signal is consistent with the hypothesis that a close association between organisms (e.g., insects and their endosymbionts) facilitates HGT. We hypothesize that these horizontally transferred genes (HTGs) play a nontrivial new role in insect immune function.

## Results

### Genomic Searches Identify Microbial Toxin Genes in Cecidomyiidae Genomes

Each of the cecidomyiid species listed above (*Co. nasturtii*, *M. destructor*, *Si. mosellana*, and *Ca. subobsoleta*) had genomic reads and assembled contigs available. We generated a shortlist of HGT candidates by excluding top matches to canonical insect genes, hits <50 AA long, duplicate or redundant hits, and hits on short scaffolds. (For more information, see Materials and Methods section.)

Microbial contamination of genome assemblies can be mistaken for HGT (Koutsovoulos et al 2016). However, there are several lines of evidence that can favor HGT over contamination (described in supplementary text, Supplementary Material online), and many of these criteria are met for the candidate HTGs we identified (see table 1 and supplementary file S1, Supplementary Material online). Additionally, none of the identified HTGs had a Shine–Dalgarno sequence, a common bacterial motif whose absence has previously been used as evidence in favor of HGT (Shine and Dalgarno 1974; Acuña et al. 2012).

**Table 1:** Final List of HGT Candidate Genes from Sequenced Cecidomyiid Nuclear Genomes, Including Information About Criteria used to Distinguish HGT from Bacterial Contamination

Species	Protein Name	Scaffold Size	Eukaryotic Genes on Scaffold	PCR Linking Gene with *Bona Fide* Eukaryotic Gene	PCR of Gene	Transcr.	Introns
*Co. nasturtii*	AIP56	10,587,749	Yes (annotated)	Yes	Yes	Yes	1
		661,609	Yes (annotated)	Yes	Yes	Yes	1
	CdtB	10,587,749	Yes (annotated)	Yes	Yes	Yes	2
	Lysozyme	3904986	Yes (annotated)	Yes	Yes	Yes	1
		3,904,986	Yes (annotated)	Yes	Yes	Yes	0
		3,904,986	Yes (annotated)	Yes	Yes	Yes	1
	SltxB	6,229,930	Yes (annotated)	Yes	Yes	Yes	2
		6,229,930	Yes (annotated)	Yes	Yes	Yes	2
*Si. mosellana*	Hypothetical protein	4,914,483	Yes	NA	NA	No	NA
	RHS	4,914,483	Yes	NA	NA	No	3
	SltxB	1,200,421	Yes	NA	NA	No	NA
		1,200,421	Yes	NA	NA	No	NA
		1,407,356	Yes	NA	NA	Yes	NA
		1,407,356	Yes	NA	NA	No	NA
		1,407,356	Yes	NA	NA	No	NA
		1,407,356	Yes	NA	NA	No	NA
		5,150,188	Yes	NA	NA	No	NA
*M. destructor*	AIP56	3,779,354	Yes (annotated)	Yes	Yes	No	NA
	Lysozyme	586,442	Yes (annotated)	Yes	Yes	Yes	0
	RHS	360,288	Yes (annotated)	Yes	Yes	Yes	2

Note.—N/A indicates that there was no sample, nearby proximal gene, or predicted gene. For further details, see supplementary file S1, Supplementary Material online.

### Evolutionary History and Putative Function of HGTs in Midge Species

The shortlist of HTGs almost exclusively includes toxin genes. They are *aip56, cdtB*, *lysozyme*, *rhs toxin*, and *Shiga-like toxin B* (*sltxB*)*.* Additionally, we found multiple copies of a gene encoding APSE-4 hypothetical protein in *S. mosellana*. This gene is found within the toxin cassette of the APSE genome (Rouïl et al. 2020). We excluded this hypothetical protein from further analyses since BLAST searches did not reveal any orthologs with known or suspected function.

**Fig. 1.**
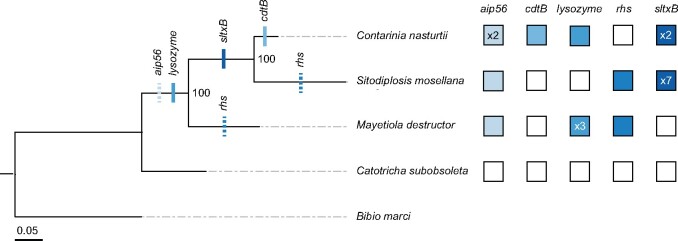
Maximum-likelihood Cecidomyiidae species phylogeny shows the approximate history of each HGT event. Filled boxes indicate presence of the toxin, and the numbers indicate copy number of the gene on *bona fide* eukaryotic scaffolds if >1 (see supplementary file S1, Supplementary Material online for additional copies on scaffolds <10 kb). Bootstrap values are reported out of *n *=* *1,000 bootstraps, and scale bar is substitutions per site. Tick marks on the phylogeny indicate approximate timing of the HGT event based on a parsimony approach incorporating presence/absence of the HGT candidate, individual gene phylogenies, and synteny data. Dashed ticks indicate HGT events for which synteny data were inconclusive.

To discern the timing and evolutionary provenance of these HTGs, we incorporated phylogenetic information and, where applicable, synteny (see fig. 1, supplementary fig. S1 and tables S3 and S4, Supplementary Material online). We used the Approximately Unbiased (AU) test to compare statistical support for the true gene phylogenies and phylogenies with forced monophyly (Shimodaira 2002). Alternative topologies rejected at the 5% significance level are consistent with the hypothesis that recipient HTG branches are from within a donor clade (Shimodaira 2002). We then used structural analysis with MAFFT (Katoh et al. 2019) and Phyre2 (Kelley et al. 2015) to help ascertain the extent to which HTGs retained their function following transfer into insect genomes. Below we summarize our findings for each of the HTGs.

#### AIP56

AIP56 is a secreted toxin of *Photobacterium damselae* subsp. *piscicida*, a fish pathogen that induces apoptosis of blood cells (do Vale et al. 2017). AIP56 is a metalloprotease A-B toxin (Silva et al. 2013), the B domain of which facilitates host cell internalization (Pereira et al. 2014). We inferred *aip56* was horizontally transferred to the *D. ananassae* species complex from an APSE-like phage (Verster et al. 2019). We previously found the AIP56 B domain encoded in a fusion gene consisting of a full-length *cdtB* gene copy and a partial *aip56* gene copy (Verster et al 2019).

**Fig. 2.**
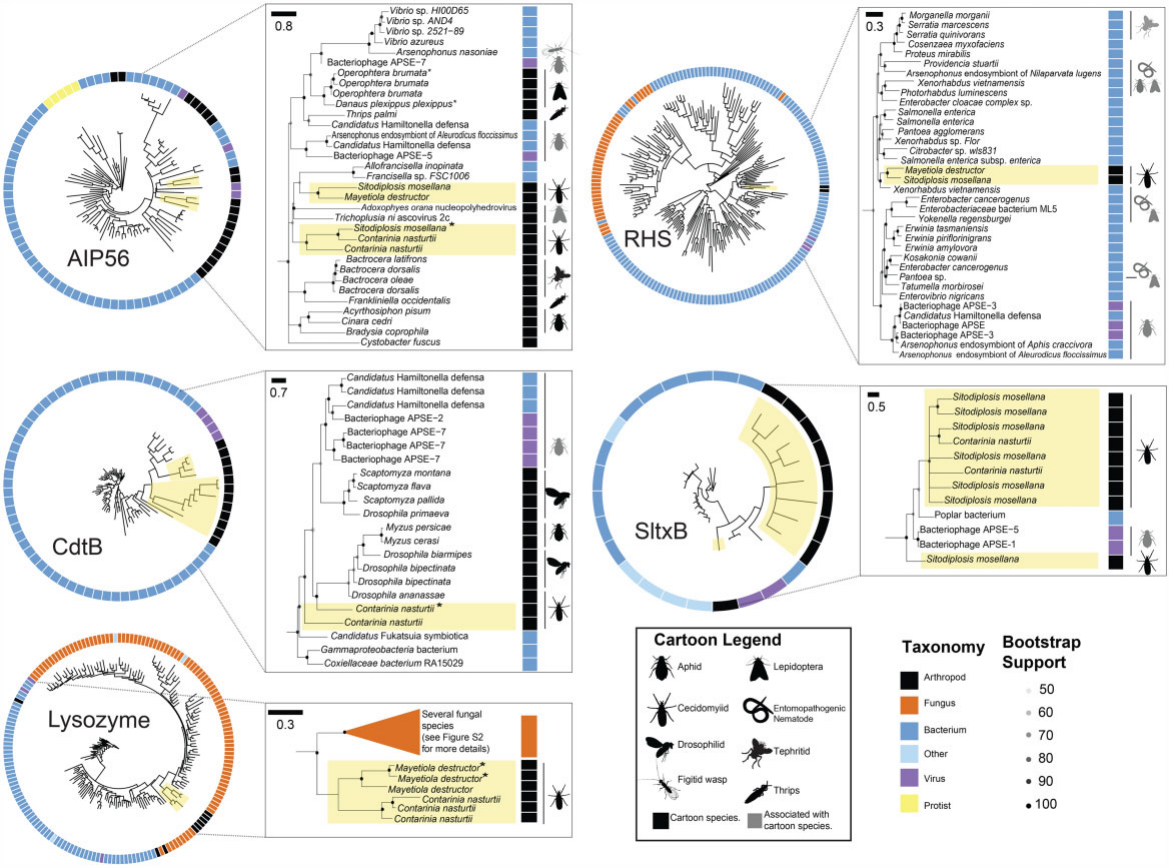
Phylogenies of horizontally transferred genes show they are nested within diverse possible donor clades, including viruses, Proteobacteria, and fungi. Indicated co-associated species suggest physical proximity that could facilitate HGT. Black cartoon organisms indicate orthologs encoded in host nuclear genomes, while grey cartoon organisms indicate orthologs encoded by taxa co-associated with the cartoon hosts. Cecidomyiid species are highlighted yellow. Possible contaminants are indicated with an asterisk. Scale bars indicate substitutions per site. For full phylogenies, see supplementary figure S1, Supplementary Material online.

Insect AIP56 protein sequences form a paraphyletic clade consisting largely of insects or insect symbiont species (fig. 2, supplementary fig. S1, Supplementary Material online). AU tests show that topologies in which cecidomyiid AIP56 are forced to be monophyletic with *H. defensa* or APSE sequences are highly unlikely (*P* = 3e−05), suggesting ancestors of neither lineage were the donor. The donor lineage may have been another insect-associated virus or bacteria, such as an ancestor of the Lepidoptera-associated *Trichoplusia ni* ascovirus. It is possible that *aip56* was transferred from the same source within insects (*P* = 0.148) and cecidomyiids (*P* = 0.753). Given the lack of clear synteny (supplementary table S3, Supplementary Material online), it is difficult to determine if HGT of *aip56* occurred once prior to the divergence of *M. destructor* and *Si. mosellana* + *Co. nasturtii* ca. 105 mya (Dorchin et al 2019), or multiple times following this split (fig. 1).

As in previous studies (Silva et al. 2013; Verster et al. 2019), we did not find conservation of the zinc-binding motif HEXXH in insect or insect-associated sequences, so catalytic activity is likely absent in insect AIP56. Short domains necessary for cellular uptake of the toxin are conserved in the AIP56 B domain (Silva et al. 2013; Pereira et al. 2014; Verster et al. 2019).

#### CdtB

CdtB is a DNase I enzyme encoded within the genomes of diverse Actinobacteria, Proteobacteria, and APSE phages (Degnan and Moran 2008; Jinadasa et al. 2011; Verster et al. 2019). CdtB complexes with Cdt subunits A and C (forming the CDT holotoxin) to enter eukaryotic cells, after which CdtB nicks the DNA, triggering mitotic arrest and apoptosis (Jinadasa et al. 2011). However, only CdtB is necessary for DNA damage and subsequent apoptosis (Jinadasa et al. 2011). In aphids, CdtB is implicated in resistance to parasitoid wasps (Oliver et al. 2009), and it may have the same function in drosophilids (Verster et al. 2019).

Since we found *cdtB* only in *Co. nasturtii*, we infer that it was introduced into the genome after the split with *Si. mosellana* ancestors ca. 70 mya, although this is a preliminary inference owing to the paucity of gall midge genome sequences (Dorchin et al. 2019). *Contarinia nasturtii* CdtB is monophyletic with respect to CdtB copies from other insects, endosymbiotic bacteria, and phages (fig. 2, supplementary fig. S1, Supplementary Material online), consistent with our previous study (Verster et al. 2019). A test forcing monophyly of insect CdtB is as likely as the actual CdtB phylogeny (*P* = 0.306), which is in turn as likely as a phylogeny forcing monophyly of APSE, *H. defensa*, and cecidomyiid sequences (*P* = 0.299). This suggests that cecidomyiid CdtB, and insect CdtB more generally, originated from ancestors of APSE- or *H. defensa*-like taxa.

Amino acid residues important for CdtB metal binding, DNA binding, and enzyme activity (Jinadasa et al. 2011) were conserved in CdtB from *Co. nasturtii* (supplementary fig. S2 and table S5, Supplementary Material online**)**. Conservation of these residues in a broad sampling of bacterial and insect taxa predicted conservation of DNase activity *in vitro* (Pons et al. 2019; Verster et al. 2019). DNase function may therefore also be conserved in cecidomyiids.

#### Lysozymes

Lysozymes hydrolyze glycosidic bonds in peptidoglycan, a component of bacterial cell walls. Lysozymes play diverse roles including in immune defense, bacterial digestion, bacterial cell wall synthesis, and release of mature phages from infected bacterial cells (Van Herreweghe and Michiels 2012).

The cecidomyiid lysozyme sequences (*M. destructor* + *Co. nasturti*i) are nested in a highly supported monophyletic clade sister to the fungal phyla Ascomycota and Basidiomycota, and distant from APSE lysozyme sequences (fig. 2, supplementary fig. S1, Supplementary Material online). This fungal lysozyme clade is sister to a large clade of lysozymes from Proteobacteria, consistent with the finding that GH25 lysozymes have been transferred indiscriminately across the tree of life from Proteobacterial donors (Metcalf et al. 2014). The AU test results suggest that cecidomyiid lysozyme genes were transferred from fungi, rather than from ancestors of APSE phages or *H. defensa* (*P* = 1e−004) (supplementary table S4, Supplementary Material online). It is also feasible that the true donor lineage has not been sampled, or has gone extinct. Lysozyme sequences are also present in the insects *Dermatophagoides pteronyssinus* and *Bradysia coprophila* and could have originated from a similar ancestral donor (*P* = 0.445) (supplementary table S4, Supplementary Material online).

The three *Co. nasturtii* lysozyme copies lie in tandem in the genome (supplementary file S1, Supplementary Material online), and there is some evidence of synteny with one of the lysozyme copies in *M. destructor* (supplementary table S3, Supplementary Material online). Maximum parsimony suggests a single *lysozyme* acquisition event prior to the divergence of *M. destructor* from *Si. mosellana + Co. nasturtii* ca. 105 mya (Dorchin et al 2019), which was subsequently lost in *Si. mosellana* (fig. 1).

The cecidomyiid and fungal lysozyme sequences share high structural similarities with phage lysozyme GH24 (supplementary table S5, Supplementary Material online). Many residues vital for binding and catalysis (Shoichet et al. 1995) are highly conserved between insect, fungal, and phage lysozyme sequences (supplementary fig. S2, Supplementary Material online). In lysozymes, conserved residues manifest in conserved antibacterial function, even following HGT between highly divergent clades (Metcalf et al. 2014). Therefore, it is conceivable that horizontally transferred lysozyme may also have antibacterial properties in cecidomyiids.

#### RHS Toxins

Rearrangement hotspot (RHS) toxins, or YD-repeat toxins, are found widely among bacteria and archaea (Jamet and Nassif 2015). RHS toxins are large and highly polymorphic, consisting of several tyrosine/aspartate (YD) repeats that are involved in trafficking and delivery of the toxin and a variable C-terminal domain that catalyzes the enzyme’s toxic activity (Zhang et al. 2012). While their function is poorly understood, they may mediate intercellular competition between bacteria (Koskiniemi et al. 2013) and possess insecticidal activity (Busby et al. 2013).

The cecidomyiid RHS proteins form a single clade sister to *Xenorhabdus vietnamensis*, a symbiotic bacterium of the entomopathogenic nematode *Steinernema sangi* (Lalramnghaki and Vanlalhlimpuia 2017) (fig. 2, supplementary fig. S1, Supplementary Material online). This clade, in turn, is sister to a group that includes *Xenorhabdus* and *Photorhabdus* species, which are associated with entomopathogenic nematodes (Boemare 2002; Busby et al. 2013). The more inclusive clade includes APSE phages and associated endosymbionts. There is no evidence of *rhs* synteny between *M. destructor* and *Si. mosellana* **(**supplementary table S3, Supplementary Material online), suggesting two independent acquisitions in these lineages (fig. 1). However, due to the long divergence time between these species and the sparse sampling of Cecidomyiidae, we cannot eliminate the possibility that *rhs* was acquired once ancestrally and subsequently lost in lineages where it is absent or recombined into new chromosomal locations. Phylogenies where cecidomyiid sequences are forced to be monophyletic with APSE + *H. defensa* sequences are less likely than those with the real topology (*P* = 4e−004), suggesting that the original donor was not an ancestor of these endosymbiotic species.

The cecidomyiid RHS sequences retain residues important for toxin function. Insect RHS sequences maintain the YDXXGR core repeat motif shared among bacterial RHS toxins (Wang et al. 1998) (supplementary fig. S2, Supplementary Material online). Additionally, three residues involved in C-terminal autoproteolysis, R650, D663, and D686 (Busby et al. 2013), are conserved in insect RHS toxin copies (supplementary fig. S2, Supplementary Material online). Cecidomyiid RHS toxins are structurally similar to the insecticidal *P. luminescens* Tc toxin complex (supplementary table S8, Supplementary Material online), which could suggest a toxic functional role.

#### SltxB

Shiga-like toxins (Sltxs) are ribosome-inactivating toxins (Chan and Ng 2016). Sltxs are AB_5_ toxins, where the B pentamer binds to globotriaosylceramide (Gb3) binding sites to retrograde traffic the active A subunit into the eukaryotic cell (Malyukova et al. 2009). Most cecidomyiid SltxB protein sequences form a monophyletic clade sister to an unidentified bacterium isolated from *Populus alba* trees (Crombie et al. 2018). This clade is sister to APSE SltxB sequences (fig. 2, supplementary fig. S1, Supplementary Material online), and the gene may have been originally transferred from an APSE-like ancestor (*P* = 0.473) rather than a proteobacterial one (*P* = 3e−05) (supplementary table S4, Supplementary Material online). Synteny (supplementary table S3, Supplementary Material online) between *Co. nasturtii* and *Si. mosellana sltxB* sequences indicate that *sltxB* was transferred to a common ancestor prior to their divergence ca. 70 mya (Dorchin et al. 2019). The gene was tandemly duplicated in *Co. nasturtii* and on several scaffolds in the *Si. mosellana* genome (table 1). Most cecidomyiid SltxB sequences form a large polytomy, consistent with a recent expansion (Whitfield and Lockhart 2007).

We found several motifs involved in Gb3 binding and cytotoxicity (Bast et al. 1999) were conserved in insect and bacterial SltxB copies (supplementary fig. S2, Supplementary Material online). Residues contributing to cytotoxicity, including F50, A63, and G82 (Clark et al. 1996), were highly conserved between bacterial and cecidomyiid species (supplementary fig. S2, Supplementary Material online). Phyre2 analyses show several insect SltxB sequences have retained a typical oligomer-binding fold, a typical SltxB structure (Ling et al. 1998) (supplementary table S5, Supplementary Material online). While the conservation of these structural features suggests the conservation of a toxic function, confirmation requires further analysis.

### Shared Environments Are Associated with HGT from Microbes to Animals

Our HTG phylogenies suggest complex patterns of ancestry between species. For example, in several cases, APSE-like phages were likely not the ancestral donors (e.g., *aip56, lysozyme*, and *rhs*). HGT has also occurred in several distantly related insect taxa, including aphids, thrips, and drosophilids. We noted that in all cases except lysozyme, phylogenies suggest that HGT occurred between insects and insect-associated microbes (fig. 2, supplementary fig. S1, Supplementary Material online). The intimate associations between plant- and microbe-feeding insects, microbial symbionts, and their phages may lead to HGT events because their DNA is in close proximity to the insect germline, which could facilitate the transfer of DNA into the germline nucleus. There is a dearth of reports in the literature that quantitatively assess the extent to which associations between insects and their associated microbes could facilitate HGT.

We tested this “transfer-by-proximity” hypothesis that sharing similar habitats leads to HGT by utilizing the *δ* value (Borges et al. 2019), which measures the degree of phylogenetic signal for categorical traits. A phylogenetic signal is the tendency of traits from related species to resemble each other more than species drawn at random from the same tree for a phenotype (Blomberg and Garland 2002). Higher *δ* values correspond to higher phylogenetic signals (Borges et al. 2019). *P*-values are calculated as the number of simulations in which the shuffled *δ* is higher than the realized *δ*, a strategy utilized in several recent studies (Pinna et al. 2020; Ronget et al. 2020).

Every species on our protein phylogenies was assigned a “niche” by searching the existing literature for information on where the taxon was initially isolated. Potential niches included living in association with plants, soil, nematodes, arthropods, mammals, aquatic environments, or other habitats (supplementary file S2, Supplementary Material online). We compared the *δ* value for the real trait distribution across all phylogenies with those for which the traits were shuffled across the tips. We found that the *δ* values for the real trait distributions were consistently higher than the distribution of *δ* values when the trait was shuffled along the phylogeny (table 2). Thus, there is some association between niche and transfer of genes between species. However, not all sampled tips represent HGT events, and the overabundance of vertical inheritance events could limit our ability to use this metric to test the hypothesis. To address this issue, we also calculated *δ* after removing vertically inherited tips from the phylogeny. This analysis yielded similar results, except in the case of *sltxB* (*P* = 0.11), which may be a consequence of undersampling (table 2). Our results suggest that the physical proximity of genomes (e.g., between two taxa that occupy the same niche) may facilitate HGT. Many of the species encoding gene copies closely related to our candidate insect genes are tightly associated with insects as symbionts, such as APSE, *T. ni* ascovirus, or *X. vietnamensis*. The intimate associations between these species and their insect hosts may have facilitated ancient opportunities for HGT.

**Table 2:** *δ* Values for Gene Phylogenies Demonstrate That There Is a Relationship between Ecological Niche and Horizontal Gene Transfer

	Real Phylogeny				HGT-Only Phylogeny			
	Tips	*δ*	Shuffled *δ*	*P*-value	Tips	*δ*	Shuffled *δ*	*P*-value
AIP56	90	7.41	*x̄* = 0.792 std. dev. = 0.218	<0.01*	52	4.197	*x̄* = 1.263 std. dev. = 0.727	<0.01*
CdtB	76	7.12	*x̄* = 0.819 std. dev. = 0.274	<0.01*	27	5.674	*x̄* = 1.222 std. dev. = 0.819	<0.01*
Lysozyme	172	7.581	*x̄* = 0.885 std. dev. = 0.320	<0.01*	117	3.640	*x̄* = 0.865 std. dev. = 0.297	<0.01*
RHS	188	8.37	*x̄* = 0.451 std. dev. = 0.134	<0.01*	76	3.215	*x̄* = 0.797 std. dev. = 0.421	<0.01*
SltxB	23	2.72	*x̄* = 0.876 std. dev. = 0.508	0.01*	8	4.500	*x̄* = 1.44 std. dev. = 2.401	0.11

Note.—*δ* values for both complete trees and trees for which we removed vertical descendance (“HGT-Only”) are shown. *P*-value is calculated as the number of simulations (*n *=* *100) in which the shuffled *δ* is equal to or higher than the realized *δ,* with an asterisk (*) indicating statistical significance (*P*<0.05). The mean and standard deviation of the shuffled *δ* values are also shown.

## Discussion

We found evidence of HGT of genes encoding microbial toxins into the nuclear genomes of several gall midge species. Microbial contamination was ruled out due to the location of these genes on eukaryotic scaffolds, regular read depth, validation by PCR and Sanger sequencing, and other factors (see table 1 and supplementary file S1, Supplementary Material online). Phylogenetic studies of the toxin sequences revealed that the copies found in cecidomyiid midges often were more closely related to orthologs found in microbes other than APSE phages, the taxa we used for our BLAST queries. We used the *δ* statistic of phylogenetic signal and found that cecidomyiid gene copies are more closely related to those found in microbes associated with arthropods or occupy similar environmental niches to the insects. These results demonstrate there is a nonrandom pattern of gene exchange between taxa that occupy similar environmental habitats.

While we assumed we would identify several instances of APSE-to-insect HGT, our phylogenetic analyses suggested a more diverse pool of HGT donors in Cecidomyiidae. This is consistent with other HGT events in this group, with possible donors including APSE phages (Zhao et al. 2015), fungi (Cobbs et al. 2013), bacteria (Cheng et al. 2020), and even other cecidomyiid species as possible donors (Ben Amara et al. 2017). *CdtB* and *sltxB* likely had an APSE or APSE-associated bacterial provenance based on AU tests. It is reasonable to hypothesize that for the remaining HTGs the cecidomyiid sequences are nested within their donor clades. However, given that these are relatively ancient HGT events, it is possible that donor lineages have gone extinct or have not been sampled. Furthermore, prokaryote–prokaryote HGT could further obfuscate the true origins of a donor lineage.

Mechanisms of HGT are better characterized in bacteria, viruses, and fungi than in animals (Thomas and Nielsen 2005; Fitzpatrick 2012; Keen et al. 2017; Touchon et al. 2017; Husnik and McCutcheon 2018). The segregation of the germline nucleus results in fewer opportunities for HGT that results in vertical transmission. Despite this constraint, new instances of HGT in eukaryotes are discovered regularly. Proximity seems to facilitate HGT (reviewed in Schönknecht et al. [2013] and Husnik and McCutcheon [2018]). Our *δ* statistic results quantitatively demonstrate that there is a nonrandom pattern of gene exchange between taxa that occupy similar environmental niches. We acknowledge that categorizing complex life histories and habitats into a singular niche is an oversimplification that might lead to overestimation of phylogenetic signals. Furthermore, our protocols may not eliminate all instances of vertical transfer, as vertical inheritance will often link taxa from different genera. However, our results are a necessary first step in quantitatively characterizing global patterns of HGT.

The majority of studies on prokaryote-to-insect HGT events have discovered genes involved in conferring new metabolic capabilities, particularly those that allow insects to colonize new plant hosts and adapt to existing ones (Daimon et al. 2008; Wybouw et al. 2016), or toxin-encoding genes involved in antibacterial defenses (Di Lelio et al. 2019; Hayes et al. 2020; Li et al. 2021). However, our study highlights that HGT of a new functional class of proteins, toxins that antagonize eukaryotic cells, may be more common among insects than previously known. Given that many of these horizontally transferred genes (with the exception of lysozyme) encode toxin proteins that target eukaryotic cellular components, they may have become integrated into existing immunological networks to protect cecidomyiids from attack by parasitoid wasps or other eukaryotic enemies. For example, horizontally transmitted parasitoid killing factors protect *Spodoptera* spp. from parasitoid wasp infection (Gasmi et al. 2021). The cecidomyiid species sampled in our study face parasitoid pressure from a wide number of taxa (Chen et al. 1991; Abram et al. 2012; Chavalle et al. 2018). We hypothesize that *cdtB*, *rhs*, and *sltxB* in particular may protect developing cecidomyiid larvae and pupae from parasitoid wasps, since these three genes are associated with this protective function in other insects (Oliver et al. 2009; Martinez et al. 2018; McLean et al. 2018). For example, APSE-3 secreted factors were sufficient to intoxicate the embryos of the parasitoid wasp *Aphidius ervi* (Brandt et al. 2017). The association of these toxin-encoding genes with insect protection leads us to hypothesize that they play a similar role in midges.

Our work contributes to our understanding of HGT in eukaryotes, particularly of genes encoding toxins that target eukaryotic cells. Moreover, phylogenetic analysis supports the transfer-by-proximity hypothesis of animal HGT. Further sampling of genomes across Cecidomyiidae may help pinpoint the timing of these HGT events and reveal more about the dynamics of HGT in this family; that is, if these toxins were gained independently several times or lost in particular lineages. Additional experiments dissecting the function of these putative eukaryote-targeting toxins may be a promising new avenue of research in this agriculturally important insect clade.

## Materials and Methods

### Identifying HGT Candidates in Cecidomyiids

We used HGT screening methods described previously (Nikoh et al. 2010), but adjusted to the scope of our study and the bioinformatic resources available. To identify possible HGT candidates in the Cecidomyiidae, we ran TBLASTN on APSE proteomes against existing genomic and/or transcriptomic resources for Cecidomyiidae species (see supplementary table S2, Supplementary Material online for proteomic queries and Cecidomyiidae databases). These searches were conducted throughout June-July 2020. We initially retained all hits with an E-value <0.01 for consideration as HTGs. Sequences were eliminated as HTGs if BLASTP searches of the predicted subject amino acid sequence (either the High-scoring Segment Pair, predicted ORF or whole length predicted annotation) to the NCBI nr database showed the top 2+ hits were to canonical insect genes. If hits were <50 continuous amino acids long, they were removed from consideration. Redundant hits, defined as hits where the same HTG from different APSE strains mapped back to the same genomic coordinate, were then removed. We also removed hits encoded on scaffolds <1 kb long, as these are highly likely to be bacterial contaminants or misassembled regions (Koutsovoulos et al 2016). Additionally, if encoded genes were <10% of the size of the canonical, functional protein, they were discarded as candidates.

### Quality Control for HGT Candidates

#### Identification of Redundant Genes

To determine if multiple HTGs were actually duplicates or a consequence of mis-assembly, we compared the scaffolds of gene duplicates using progressiveMauve (Darling et al. 2010). If there was >90% nucleotide identity between scaffolds, we considered those mis-assembly artifacts. If the subject sequences shared high AA identity (>90%) throughout multiple ranges on the same scaffold, we considered this as evidence of HTG duplication. These duplications were subsequently corroborated by BWA analysis.

#### PCR

For *Co. nasturtii* and *M. destructor*, we validated the HTGs with PCR and bi-directional Sanger sequencing (see supplementary methods and table S6, Supplementary Material online) of genomic DNA. In cases where the distance between the GOI and a proximal gene was <2,000 bp, we amplified regions that included other *bona fide* eukaryotic genes.

#### Synteny Analysis

Possibly due to the long divergence between sequenced species (e.g., our most related species *Co. nasturtii and Si. mosellana* are estimated to have diverged from a common ancestor ∼70 mya [{Dorchin et al. 2019}]), macro-syntenic analyses using progressiveMauve (Darling et al. 2010) and CoGe SynMap (Lyons et al. 2008) were not fruitful. Instead, we employed a qualitative micro-syntenic approach. In annotated genomes, we extracted the protein sequences of genes up and downstream of the HTG and indicated their position with −*n* or +*n* (e.g., a positionality of −3 indicates the gene is located three genes upstream of the HTG). These sequences were then submitted as TBLASTN queries (Altschul et al. 1997) to the representative genomes. The scaffolds of top hits were then extracted. If there were no hits, we indicate “NA” in the cell. We considered there to be some evidence of synteny if one or more genes proximal to the HTG were located on the same scaffold within a species. Results are shown in supplementary table S3, Supplementary Material online.

#### Identification of Bona Fide Eukaryotic Genes on a Scaffold

To determine if the HTGs were encoded on scaffolds with other eukaryotic genes, we used existing annotations (see supplementary table S2, Supplementary Material online). If the genome was not annotated, we ran Augustus annotation on each scaffold under consideration using the “fly” setting as implemented in Geneious (Stanke et al. 2004). If Augustus did not predict genes, we used Geneious v. 11.1.5 to predict ORFs >500 bp, which were then submitted to NCBI BLAST in order of proximity to the HTG. If at least one of these ORFs hit a *bona fide* eukaryotic gene, we marked the HTG as being on a scaffold with other eukaryotic genes. HTG candidates encoded on scaffolds <10 kb with no other *bona fide* eukaryotic genes were removed from our list, but are retained in supplementary file S1, Supplementary Material online.

#### BWA Analysis

We aligned Illumina reads (see supplementary table S2, Supplementary Material online for SRA accessions) to the genome via BWA (Li and Durbin 2009) to search for unusual coverage depth relative to neighboring genes, which can be due to contamination (Koutsovoulos et al. 2016). Read quality and trimming were assessed with FastQC (Andrews 2010), which showed high per base sequence quality, low per base N content, and low adapter content in the available WGS data sets. The read alignment was visualized and assessed in the software package Geneious v. 11.1.5 (https://www.geneious.com). Since the majority of the genes were encoded on scaffolds encoding other *bona fide* eukaryotic genes, we included the read depth of all candidate scaffolds, per species, in a Grubbs’ test and removed scaffolds with reading depth outliers. Following this, we did the same with the loci containing the horizontally transferred genes (HTGs), including those of all tandem duplicates. The results show there are no coverage abnormalities, suggesting the HTGs are not assembly artifacts or microbial contamination (for full results see supplementary file S1, Supplementary Material online).

#### Transcription Analysis

We submitted the GOI (+/− up to 20 kb up and downstream) as a blastn query to representative polyA-enriched transcriptomes. These representative transcriptomes are shown in supplementary table S2, Supplementary Material online. The top hits (≤5,000) were extracted and mapped back to the region using Geneious RNA Mapper (Sensitivity: Highest Sensitivity/Slow; Span annotated introns). We report the mean read depth and standard deviation across the GOI in supplementary file S1, Supplementary Material online.

#### Identification of Introns

Since many of the HTGs in the *Co. nasturtii* and *M. destructor* genomes were annotated, we used existing annotations to predict intron boundaries where applicable. If the gene (or, in the case of *Si. mosellana*, the entire genome) had not been annotated, we ran Augustus annotation on each scaffold under consideration using the “fly” setting as implemented in Geneious (Stanke et al. 2004). In the “Intron” and “Exon Coordinates” columns, we indicate the number of introns predicted by either annotation specific to the species or Augustus annotations. In some cases, Augustus did not predict any genes in the region of interest, in which case we reported “NGP” for “No Gene Predicted.” Note that Augustus relies on training on the appropriate gene sets (Stanke et al. 2004), and it may fail in cases of HGT due to the inherent differences of genes with horizontal provenance. Where the HTG does not have an associated annotation ID, we report the Augustus-predicted exon coordinates (supplementary file S1, Supplementary Material online).

### Species Phylogeny and Ancestral State Reconstruction

Nucleotide sequences for *co1*, *cad*, *ef1a*, and *28 s* were retrieved from GenBank for each of the five species included in the species phylogeny (supplementary table S7, Supplementary Material online). *Bibio marci* (Diptera: Bibionidae) was included as an outgroup to the Cecidomyiidae family, consistent with phylogenies previously generated for the family (Sikora et al. 2019). Each gene was aligned individually using the default settings on the MAFFT v. 7 webserver (Katoh et al. 2019). Individual gene alignments were inspected and manually trimmed before concatenation. The final alignment consisted of five species and a total of 3,135 nucleotide sites. Total sequence lengths for each gene were as follows: *co1* (542 nt), *cad* (1,439 nt), *ef1a* (725 nt), *28S* (429 nt). The concatenated alignment was uploaded to CIPRES web portal for maximum likelihood (ML) tree construction. An ML tree was generated using RAxML-HPC2 on XSEDE using default settings (Miller et al. 2010; Stamatakis 2014). The ML species tree is shown (log-likelihood = −10311.662040) with bootstrap values at each node (*n *=* *1,000 bootstraps) (fig. 1).

Due to the low number of taxa on our tree, maximum likelihood approaches to timing HGT events were uninformative. We opted to take a maximum parsimony (MP) approach to infer the relative timing of each HGT event by incorporating data from synteny analyses and Approximately Unbiased (AU) supported protein phylogenies. Briefly, we assumed a single acquisition of the HTG in the common ancestor if there was evidence of shared synteny among the taxa in which the HTG was found (supplementary table S3, Supplementary Material online). In the absence of synteny data, we examined the protein phylogenies to determine the relative timing of HGT events (supplementary fig. S1, Supplementary Material online). We interpret monophyly of cecidomyiid protein sequences as a single acquisition, or several acquisitions, from a similar common ancestor under an MP model. Acquisition events that are only supported by protein phylogeny data are indicated on the species tree with dashed ticks (fig. 1).

### Protein Phylogeny Construction

Representative toxin sequences were queried against the NCBI refseq protein database on November 20, 2020, using BLASTP (Altschul et al. 1997) with a maximum of 500 top hits per query (see below for a list of query sequences used per toxin). We selected query sequences that represent major insect or endosymbiont clades for the GOI. Top hits were extracted for each sequence. For clarity, redundant sequences were removed with cd-hit (Li and Godzik 2006; Huang et al. 2010) with a 0.8 similarity cutoff, unless they were genes specifically identified in this manuscript. Synthetic constructs were manually removed.

Sequences were aligned with MAFFT v. 7.312 using the E-INS-I strategy and the BLOSUM62 amino acid scoring matrices (Katoh and Standley 2013). Sequences were trimmed to include only the conserved protein domains (i.e., domains in which <50% of the sequences had gaps). After trimming, sequences were re-aligned with the earlier MAFFT settings.

Gene topologies were inferred using maximum likelihood as implemented in W-IQ-TREE (http://iqtree.cibiv.univie.ac.at/) (Nguyen et al. 2015; Trifinopoulos et al. 2016) using the best-fit model as assessed by BIC in ModelFinder (Kalyaanamoorthy et al. 2017). The resultant consensus tree was constructed from 1,000 ultrafast-bootstrapped trees (Hoang et al. 2018). Nodes with <50% bootstrap support were collapsed to polytomies using the di2multi function in ape v5.4 (Paradis and Schliep 2019). Phylogenies were visualized and annotated using ggtree v. 2.5.0.991 (Yu et al. 2017; Yu 2020). Specifics of each phylogeny are shown in supplementary table S8, Supplementary Material online.

Note that in the cases of *aip56, cdtB*, and *lysozyme*, the genes of interest appear to have been transferred into insects besides the cecidomyiids investigated in this manuscript. We evaluated whether these additional putative HGTs are contaminated in supplementary table S9, Supplementary Material online. For each case we examine the size of the scaffold on which the gene is encoded, determine if there are other *bona fide* eukaryotic genes on the scaffold, and, if there are multiple species from the same genus, evaluate if the gene is syntenic (e.g., *Bradysia*, *Bactrocera*). We do not evaluate drosophilid or *Myzus cdtB* for contamination as this has been previously done (Verster et al 2019). In only one case (*Aphis gossypii cdtB*) is there strong evidence that the HTG is actually bacterial contamination.

### Evaluation of Phylogenetic Topologies

The alignments from the above methods were used for the evaluation of different topologies. Parameters of the phylogenies were set in BEAUTI v1.10.4 (Suchard et al. 2018). Several topologies were created: the actual topology, or topologies such that listed clades were forced to be monophyletic. Forced monophyly was accomplished by selecting the -Mono? and -Stem? options on all selected sequences in BEAUTI (Suchard et al. 2018). Specifics of forced monophyly are shown in supplementary table S10, Supplementary Material online. Substitution and site heterogeneity models per phylogeny were selected from supplementary table S8, Supplementary Material online. Phylogenies were built using BEAST v1.10.4 (Suchard et al. 2018) on University of California—Berkeley’s Savio HPC cluster, running Markov chain Monte Carlo (MCMC) for 10 million iterations. Following phylogeny construction, chains were analyzed for convergence with Tracer v1.7.1 (Rambaut et al. 2018). Postburnin samples (10%) were used to construct maximum clade credibility with mean node heights in TreeAnnotator v1.10.4 (Suchard et al 2018). FastTree (Price et al. 2009) was used to reoptimize branch lengths and report Gamma20 likelihoods for fixed topologies (settings: -gamma, -nome, -mllen). The perl script GammaLogToPaup.pl (http://www.microbesonline.org/fasttree/) was used on these reoptimized trees to reformat the information for use with CONSEL v1.19 (Shimodaira and Hasegawa 2001). P-values from the AU test (Shimodaira 2002) are shown in supplementary table S4, Supplementary Material online. Alternative topologies rejected at the 5% significance level can lend support to the hypothesis that the recipient HTG branches from within a donor clade.

### Measuring Phylogenetic Signal

For all species in a phylogeny, we assigned a “niche” trait that fell under *Arthropod*, *Plant*, *Nematode*, *Mammalian*, *Aquatic*, *Soil*, or *Other*, assignments that were meant to broadly describe the species niche*. Other* included other habitats that did not fall under the named categories. To assign these categories, niche information was taken about the isolation source of the genome in which the protein was annotated on NCBI GenBank. If there was no information on GenBank, we searched JGI IMG/M (Chen et al. 2019) or JGI MycoCosm (Grigoriev et al. 2014). If neither of these was fruitful, we last searched Google Scholar for peer-reviewed, primary literature about the strain of interest. If this approach still yielded no information or contradictory information, we indicated *Other*. Niche assignments and corresponding citations for tips are shown in supplementary file S2, Supplementary Material online.

We utilized Borges’ *δ* value to evaluate the phylogenetic signal of the species’ niche traits (Borges et al. 2019). The value of *δ* can be any positive real number. The higher the number, the higher the phylogenetic signal (Borges et al. 2019). This can be compared with the *δ* value of the same tree with randomized or shuffled traits to assess significance. To determine whether to “shuffle” traits (i.e., re-arrange the traits) or randomly assign traits, we piloted this analysis with both and found that the shuffled trait set has a higher *δ* value, and as such is a more conservative method that we continued to implement.

We calculated the *δ* value using lambda = 0.1, se = 0.5, sim = 10,000, thin = 10 and burn = 100 in R (R Core Team 2017). The originally calculated phylogenies were used, except without the utilization of the di2multi() function in ape (Paradis and Schliep 2019). To determine whether the realized *δ* value is statistically significant, we randomized the trait *n *=* *100 times along the phylogeny and calculated *δ* for each shuffling using the replicate() function in R (R Core Team 2017). The real value was compared to the randomized distribution of *δ* values. P-value was calculated as the number of simulations (*n *=* *100) in which the shuffled *δ* is higher than the realized *δ*.

To improve the robustness of our conclusions, we removed, to the best of our ability, vertically inherited tips from our phylogenies. We used the drop.tip() function in ape v5.4-1 (Paradis and Schliep 2019) to manually remove paralogs from the same genus (or the same family in the case of cecidomyiids). Paralogs were removed if they were both in the same highly supported (i.e., >75% bootstrap support) ingroup, or a paralog was a singular (i.e., without a sister taxa) outgroup to the clade containing the other paralog from the same genus. Tip trimming was done with no prior knowledge of species’ niche. This process was repeated iteratively until the final trimmed tree had no sister taxa from the same genus. We show trimmed, HGT-only trees in supplementary figure S3*a–e*, Supplementary Material online, and an illustrative example of how the tip trimming was executed in supplementary figure S3*f*, Supplementary Material online. We calculated the real and shuffled *δ* values as described above on the pruned tree (table 2). *δ* and p- values for both the actual and HGT-only trees are reported in table 2.

## Supplementary Material

Supplementary data are available at *Genome Biology and Evolution* online.

## Supplementary Material

evab202_Supplementary_Data
