# Supplementary material for: Horizontal Transfer of Microbial Toxin Genes to Gall Midge Genomes
Source: Genome Biol Evol. 2021 Aug 27;13(9):evab202. doi: 10.1093/gbe/evab202 (PMC8455502; doi:10.1093/gbe/evab202)
Supplement: evab202_Supplementary_Data [file evab202_supplementary_data.zip › REV2_SUPPLEMENT.pdf]

Supplementary Methods

gDNA extraction and PCR conditions.

Ethanol-preserved samples of *Co. nasturtii* larvae and adult males and females from a lab-reared colony at University of Vermont (courtesy of Dr. Yolanda Chen and Andrea Swan), and ethanol-preserved samples of *M. destructor* larvae and pupae were provided from a lab-reared colony at Kansas State University (courtesy of Dr. Ming Chen). *Sitodiplosis mosellana* only have one generation per year (Elliott et al. 2011), and we were not able to obtain gDNA for this species.

Specimens were rehydrated in sterile water and allowed to dry on a Kimwipe. Rehydrated specimens were homogenized with a bead-beater for 2 minutes at 30Hz. DNA was extracted from the homogenized samples using a DNEasy Kit (Qiagen) with an overnight Proteinase K digestion at 55˚C.

We designed PCR primers to capture the HTG and, if the nearest gene was <2kb distant, a neighboring *bona fide* eukaryotic gene. PCR primers were designed using Primer3 as implemented in Geneious v11.1.5 (https://www.geneious.com)**.**

PCR reaction mixes were either done with *Taq* polymerase (New England BioLabs) for *Co. nasturtii* samples, or Phusion ® High-Fidelity DNA Polymerase (New England BioLabs) for *M. destructor* samples.

Taq PCR reactions were composed of: 7.5µl Failsafe Premix E (Epicentre), 4.2µl nuclease-free water, 1.2µl each of F and R primers (IDT), 1µl template DNA, and 0.12µl *Taq* polymerase (New England Biolabs). Thermocycler settings were: 5 m at 95°C and 35 cycles of 95°C for 30 s, Ta for 30 s, and 68°C for 1-2.5 m depending on amplicon size (see **Table S4**), followed by a final 5 m extension at 68°C.

Phusion PCR reactions were composed of: 8.4 µL nuclease-free water, 3 µL Phusion HF buffer, 0.3 µL 10 mM dNTP mix (Invitrogen), 0.75 µL F and R primers, 0.45 µL DMS), 0.15 µL Phusion polymerase, and 1 µL of template DNA. Thermocycler settings were: 30 s at 98°C and 35 cycles of 98°C for 10 s, Ta for 30 s, and 72°C for 30 s to 50 s depending on amplicon size (see **Table S4**). The first five cycles were run using a touch-down starting at 10°C above the Ta until lowered to the final, indicated Ta. The run ended with a final 10 m extension at 72°C.

1% agarose 1X TBE gels were prepared with Apex Agarose in 1X TBE buffer with 1 µL SYBR™ Safe staining gel per 10 mL of gel solution. 4 µL of PCR product was mixed with 1 µL ThermoScientific 6X Loading Dye. 1Kb Plus DNA ladder (Invitrogen) or 100bp DNA ladder (NEB) was included as a molecular marker for *Co. nasturtii* and *M. destructor*, respectively. PCR product was run on gels using the Owl™ EasyCast™ B1 Mini Gel Electrophoresis System rigs for 30-40 minutes at 120V. Gels were visualized using AlphaImager™ Gel Imaging System (Alpha Innotech). In some cases, vertical white bars are used to indicate different images from the same gel/primer were stitched together for clarity; besides the concatenation these images were not subject to other nonlinear adjustments.

Polymerase used, primer sequences, the region captured by the amplicon, melting temperature, extension times, and the expected amplicon length are detailed in **Table S4** along with gel images. All PCR products were Sanger sequenced in both directions at the UC Berkeley DNA Sequencing facility.

Structural analysis.

To model and predict protein structure and function for representative proteins, we used the Phyre2 web portal (Kelley et al. 2015) using the “Normal” modeling mode (**Table S5**).

To determine at the residue level the extent to which vital catalytic residues are preserved in disparate lineages, we used the MAFFT aligner as described above with representative sequences (Katoh & Standley 2013). The IDs for representative sequences shown in **Figure S2** are indicated in **Table S11.** Note that due to the low conservation between AIP56 insect and characterized sequences, we did not show this alignment. Alignments shown in **Figure S2** were visualized using BoxShade (https://embnet.vital-it.ch/software/BOX_form.html).

Supplementary Text

Criteria used to distinguish true HGT events from possible microbial contamination

We present these in ranked order of importance.

1. PCR evidence links the GOI to another *bona fide* eukaryotic gene. The generation of an amplicon that includes both an HGT and a canonical eukaryotic gene would not be possible unless they are proximal in a genome.

2. The gene is syntenic in two or more species. This demonstrates vertical inheritance following an initial horizontal transfer. While HTG synteny in a lineage can provide strong evidence in favor of nuclear integration, the under-sampling of cecidomyiid species (e.g. the closest related species, *Si. mosellana* and *Co. nasturtii* are ~70 my diverged [Dorchin et al. 2019]) meant that synteny was often difficult to assess.

3. The gene is encoded on a scaffold with other *bona fide* eukaryotic genes. This indicates the gene is not only present (which could easily be indicative of bacterial contamination), but shares the same high-quality scaffold as a canonical eukaryotic gene (Husnik & McCutcheon 2018).

4. Burrows Wheeler Alignment (BWA) shows that the coverage for the GOI is not irregular, i.e. is not far higher or lower than that of other genes and/or scaffolds in the genome assembly. This line of evidence is similar in premise to that above.

5. The GOI is transcribed in dT-enriched transcriptomes. In eukaryotes, poly(A) tails usually stabilize intact mRNAs, while in bacteria such as *E. coli* poly(A) tails are targeted for degradation (Dreyfus & Régnier 2002). Thus, it is unlikely poly(A) enriched gene fragments will be present in significant quantities in bacterial contaminant genomes. We do not consider absence of transcription evidence against HGT, as transcription of a gene will be heavily contingent on the tissue type and life stage of the organism.

6. The GOI is predicted to have introns, which are rarely present in bacterial genes. Intron predictions are far stronger when generated with data from dT-enriched transcriptomes, since the alignment of the transcript to the assembly sequence reveals intronic boundaries. Intron prediction using programs such as Augustus are often based on homology (Stanke et al. 2004; Keilwagen et al. 2016), which may be difficult to utilize in the case of horizontally transferred genes since bacterial or viral genes are not typically intron-bearing.

7. The gene of interest was amplified via PCR This shows that the gene appears in another sample besides that of the genome assembly. However, this could also theoretically be explained by the gene being encoded in the genome of bacterial symbionts.

8. Other predicted signatures of eukaryotic domestication, such as initiator sequences, UTRs, and TATA box motifs (see below). It is possible these motifs could appear by chance, and not all eukaryotic genes have all of these eukaryotic motifs.

Domestication of various bacterial toxins following horizontal gene transfer from prokaryotes to eukaryotes

The presence of eukaryotic transcriptional motifs in putative HTGs may indicate adaptive optimization for a gene of prokaryotic origin in its novel eukaryotic context. Here, we analyze HGT sequences for motifs related to eukaryotic transcription, largely following methods described in (Verster et al. 2019). Briefly, we analyzed the regions flanking our candidate HTGs for core promoter elements identified by transcription initiation factors TFIID and TFIIB (summarized in (Thomas & Chiang 2006)), alternative transcription initiation elements such as the GC box (Blake et al. 1990) or CAAT box (Graves et al. 1986; Raymondjean et al. 1988), and transcription termination elements such as polyadenylation signals, cleavage sites (CA), and upstream and downstream sequence elements (summarized in (Proudfoot 2011)). We also searched the sequences for the Shine-Dalgarno sequence, a motif essential for bacterial ribosome binding (Shine & Dalgarno 1974), which can indicate that our HTG may be a bacterial contaminant, as well as motifs for eukaryotic translational start sites, like Kozak sequences (Cavener 1987). This list is not exhaustive, nor will every element described above necessarily be found in all eukaryotic genes (Kutach & Kadonaga 2000).

We did not analyze candidate HTGs from *Si. mosellana* since the genome was unannotated, making it difficult to accurately predict gene boundaries. Additionally, we did not analyze the horizontally transferred lysozyme copies, since phylogenetic analyses indicate these were transferred from a eukaryotic donor (see **Main Tex**t).

Co. nasturtii

Legend:

- Predicted exons are highlighted in blue and predicted introns are highlighted in yellow. Exon/intron boundaries for *Co. nasturtii* are taken from the GenBank assembly annotations.
- Coding sequences are indicated in bold text. 5’ and 3’ UTRs are therefore designated by unbolded text highlighted in blue.
- Poly(A) signals or cleavage sites are underlined. Upstream and downstream sequence elements are italicized.
- Intergenic regions (between *sltxB* copies) are designated by lowercase text.
- TATA box motifs are designated in orange. Initiator sequences are highlighted in white text when found within annotated mRNAs, or in blue text if found outside the gene boundaries. Kozak sequences are highlighted in green.

cdtB on NW_022197544.1:

TCGAAATTTATTTTTTGTTTTTGCTTTTCATTACATTTTCAGACATTGAATTGTGCGTGTATCT
TTTTGTGTGTTGGACAAAA**ATGAAGGGAGTTCTATTTTTTGGATTCATGTGTGCAACATTCATG**
GTAAATTTTAAATATAAAACTTAAATGTTCATTCCAGTTTGAAATTTCAATATTTGCTTTTTTT
TTTGTTTTTGTTCAG**AATTGTTATGGAAGGGTTTGTAGTGATATGGCATATGATATCCCGATGG**
CTACGTGGAATAGTCAAGGCGGAAGATGGGGTACGGTGAAAACATTGTTATCACATACGTATCC
TGATGTCGAGGTACTTGCATTGCAAGAATGCGGCAATCCGCCTATTGATCCAGCTATAGCACTT
GTTGGAAATGGTAATATACCAACACAATGGTCTGATAATAGACCATATTTGACTGTGCAGTGAG
TAAAACTTCTGTTGCATTTTTATTTATAAATTATGTTGATAACTATAAGTGAATCTAACTAATA
TAAAACAATTTCTATAG**ATATATATCCTATTTCTATAATAATGGTGATATTAATCAAAGAGATA**
ATGGAAGTGGCGCAAAGGAATATACTATCAATGTGAGAGATAGAGGCCATGTTCAAAGAATATA
TTATTTATATCATTACGAATTACAATTGGCTGGAAATGTAAGAACAAATACTGCCATAATCACG
AAAACAAGAGCGAATGAAGTGTTTGTTTTAATTGATCCAAATGAATCAAGACCAGTAATTGGCT
TCCGAATTGGAAGTAATTATTATTTTAGTATGCATGCTGGGGCTTATCCGAAAAATCCTTCTCC
AGATACAGTCTCACAAATTGCACAATTTGTTAGCAACAACGCAATGCCTATGGAAGACGTTTCA
TTTGTAGTAATGGGCGATTTCAACACAGAGCCTAATTATTTTAACCCCACCGTAATACCTCGGG
GTTTCCATTTTACAAAAGTTCTACCATCTGAGAAAACACAAGGTCTTGGCAATACTGTAGTTAG
GTTATACGATTATGCATTTATTGGAACAAGAAACACTTGTGAATTTCCAAATTTTATTGTAAAC
GCTGGGAATAAGTACGACAGTGATCATCGAGTTGTTGTATTTCGAAGACAATAGCAAG*TTTTAA*
GCATAGGACTGGACTAATAAACGTCAATAGGGGTTGTCATTC*TTGTTT*AATCAATTTACTTCAA
ATTTTCAATAAAGAAAAAAATGTTTCAAA

cdtB on NW_022203704.1:

TATAAATATGAACTTTATAATTTTGTGAAATCATTCATATTTTGAATGTCGAATTGTGAATCGA
TTCTCAAATATGCAAGGGAAATTAAAACGATCAGTGTTATTATTGTCGAAGGAATAATTCTATC
AAGCATCAAATATTAGCATTGCGAGAGTTAGTATCAAGTTATTTTGAAGAATTTTGGGAAAATT
AACTACCACACAAAAACAGATGTTCGATGTTGAAGAGTATAATAATAATTTAATCCTTTATAAT
TATTTCAAATATCCACAGAATAAAT**ATGAATACTGAGGTCAGCGATTTTGTTTTCGTTACATGG**
AACACCGAAGGTTCCAATTGGGAGAATGTCGCAAAGTTGATGCTCAGCAAAGAAGGTGTAGACC
GAATCGATGTTATGGCTCTTCAAGAGTGTAGTGAACATCCCATCTCTGATGAGCATCCACAGGC
GGCAAAATCAGGTATTGGAACCATATCTCTACCTGCCAATGAAGTAGAAAATATTCAAATAAAT
CCTACACCGAATCAACGAGACAATTCTTCAGGAATCACTACCTACCTATGGCATTTCAACACAG
AAGCAGAGTTTTTTTTGTATTATCGAAACAACAAGATATTTACTGAAATTGGTGCAGTTGGTGG
ATCAGGAAAAAAGAGCCTGAGTAGTGCCTTTGTAACTAGAGTCGAGGCTACTAGAACTTTTTAC
ATGGCTCCAGTCGATAATAACGGTAGTCCAGTAAATAATAGTGACTACAATAAAAACCGTCCTG
TTATTGGGATTGAGGTGAATGGTGTCGTGTATTTCAACATTCATGGGTCGCATCAGTATAATAA
TTCTGTAAATAATACCATTAGAATTATAAAAGAGTTTATGGCAAGGAACCATCCTGCAATAAAA
TGGGTAATGATGGGTGACTTCAATAAAACGCCACTAGAGATTAATACTGCGGGATTGTATTTGA
TGGAACCAAACACAGCCACTCGTGCTAAGAGCGGTAAGATAATTGATTATGCCATATCTAATGA
TTCAACCGTTGGGAATATGCATATACATGTTTCACGTGACAATCATGGATCAGACCATTTTCCA
GTGGAATTTATCAATAAAAATATAAACCACAACAGTTTTGGATCATTTTAGCTTTCATTAATGA
CGGCACTAAAGAAGCCATAAGAAAACCATAGA*TTTATT*GCCTAAAACGATAAAAAATAAAATTC
ACATATAGTTGGCGCTGCATTCTATGCAAAC*TTGTTTT*CCGTTGCA

aip56 on NW_022197544.1:

TTTTTTTAAATTTCGCTTGCTTTTGAGAAAGAAAAAATATTATAATATAGTGTTTGATTTATAC
GGAAACATTTAAAA**ATGTTAAAATGTATACTTTTGGCTCTTGCATTTACGCATTTGTCTCAAAC**
CAACTTTTTAGATAGCGGTAAATTGTTTATTTTTTTCCAAATTTTATTGAATTTTTATATAATT
TGTTATTGTGTACGTACAACAG**GTCCATTGGACTGGACTTGCATGTCATGGCTTGCCGGTTCAC**
GGAACAGGCCGTCAGTTCAAGATTCTACTTTTTATAACTTGGTGCACAATATTCCTTCTACAAG
AAATGCAGATTTACACTTCTCATTATCGCGACATCACATAATACCTTATAAAGTGTTATATAAA
TTTTTTAATACCGTATTGGAGTTGGGTGAATCTAACTCAAGAATTCATTTTTTGCTTGGGCAAT
TCTTGTCGAATCTACTTGTCGATTTAATGGTTCGCGCTCCCGGCGCTCAAGATCAGACGGAAAC
CGAAGTTTTACAATCCATTCGCAATTTATTTCAAGGCTTTGATGGTGCTGGATTTGGTGATACA
GTGCAGGGAGAAATCCGAACATTTCGTTTACCGAACTTTCAAGAAAACATAGTGGCATTCCGTC
ATCTACCGCTCACCGAGCTAACAAATACACAACTCGTAAGGAATATTGCTCAGGTCAGAATTCA
AATGACACTCCAATCTCTATTGACATGGATGCCATTTAATTATTTTGAGGGGCCCAGTGGAATG
TATCGTACCGACGAACCCGGTCCAAATTTTGAAGATAATGCGTATGTAATAATCGGTGAAGAAA
ATTCGGTTCGACTACATGACGCATACGAAACAATGATTGCAATTGATGAAATACGGACGCAGCA
TCCGACAATTATTCCGTTGCAATCTATACAATTTGTATTTGACAATTTAAATGTAGTTCGAGGA
AACGCACCTAACGGATATGGGCAATTTAACCTGGGATATTGGGAAGAACTCGAACCGGTTGAAA
AGAAAAAAGGTAAATTGGAATGTCAATTCAAAATCCAAACAACAAGACCAACAACCACGCCACC
GCCCCATCACAATCACCGACCTCATGAGGAATTCCGTCGAAAAAACAAAGTTTCTTCAGGAACG
AACAGCTTAGACCCGCTTTGTTTAAATACAACGTTTGAGGCTATCAAACTTTATACTGATAATT
TATATTTAGCCTCTAAACCTGGTAAAGTGCCCAATGTACATCAATACATATGTAGAGGAATATG
GGAGAGCTATTTAGCAGCTTTTGGTATATCGCAATGTCTTCCGTAATACTATAATTTTTTGAAT
CACCATATATGGCACGTGAATTAAAACTTTTCTTCTTTCAAAATCCGTAAATTTTTTTTAAAAA
ATCCTTAGTTAGTAAAAATATATGAAAAGAAATTGAATTGGACGTTCTCGCTTTTTGAACATAT
ATAAACTGGTTGGTAGTTAGCTATGGAAATTAGTTATGGGTTGCAACGTATTACATTCGCAATT
AGATTTTTAGAAAAATTTATTGAACAACGAATCTCTTAGAACTACAATCGGATTCTTAAATCTC
ATGTTGTGCTTTTAAATTCAACGAAACTATCAACTATTGATCATTTTAAACACAATCAAAATAG
TTTTCTGCTCATTTTAATTAACATTTTATACTTAATCATCATGTTTTAGACATTTATTAAACAC
TAGGCAAACAAACTTAAGTTTTTAAGATGAATTCATTTTTTTTTCTTTTCATGTGTTCACTCCAA
CGAGATACATTTTAAAATAAAGAATTTTATTGAGTTTGAATGCTATTTTCTGCTCA*TTTTTTAT*
*TGTTTTTGTTTTTTGT*ATCGCTTTTGATGGAAAAAAAAATAGCATTGAAGAGGATTTAATTG

aip56 on NW_022200251.1:

TTCGAGCAAGAGTTATCAATATCAATACATTAGAA**ATGATTAGACTTACATTGCTTCTGATTTT**
CGTGGCTATTCTCTCTGCGGCTCGTGAAATCGAACAGTAAATTCTAGTTTTTGTTTTCGCTTTG
TTTTGATAATTGAATATTTTTACATTTTAACGATTACGACATATTTGTAAGGCGTATATATATG
ACATAGTACATCGTATTTAGTATCGTATAAAACCATAAATTTCCTTCGCCGTGGATTCACTGCT
TCATTGCCGTTTTGAACTAGTTTTGTTCACATCAAAATTTATCTGTAGAATCTAAATAATGGTA
TGTGTGAATGTGTTTCATAGAACAAATACTTCGTGACAACAGAAATAAGTTTATTCGTTGAAAG
ATAAAACTTCAGATTTTCGTTGCGGAAGCGAATGAATAATTTTTAATTCAAATAAAAAATTGCT
CGTGATTCACATATCTTATCGACTTTTCTTCAATTCAAAAGTTACGATGTTATTTCTATAAAGC
CATGTTGATATATATTTTCTGTTTAGGTTAATCGTTATATAAATTCGATCCGAAGTCAATGTTT
GATAAAGAGTCTTTTCAAACTATCTTTTTTTCCATAGGTAGTTTGAATATTTCGATATTTTCTT
TTGACATTTTGGCTCTCCCCAATTTTGACACTGAAATAGTCTAAGGAACTTGATTTTTGTTTCA

AAGTAAAAGTATGAAATTTATTCAATATATTAAATTTATTTGTTTTCAGAAG**TATCGAAGATGA**
ACCGTGTACGTATGACTTGGCGCGTCATCATATCATTGCATACAGTAAGGTGAAAGAGTTTTTC
GAAACAGCCGCTGTGAATGTGAAAAATCGTGAATTACGACGGCGATTGGCAAAACTATTTGAAA
AATTAGCGACACATCCAAATGAACCAATGAACGAAGACGACCACACTAGTCTGTTCGAACGAAA
TGCATTCGAAAAGGAGTTATCAACACCGTTGCAGTTACTTGGAATTGGCAGTTATGATGCGAGA
AGAGTGGCTATCTCGCTGATTCGATGGATACCATTTAACATTTTCAAGGGTCCTGCAGCTAAAA
ATCGAGTTGATGATCCAAAAAATGGATTCGAAGAAAATGCTGGCCGAATTGTAAATGCGGACAC
ACGTGACAATTTAGAAAATTTGCACATTTTATATGATAACATGATAAATTATGTCAACACAGAC
AGTGTGGATAATTTCAAGAATTCAATTAATTTAATGGAACATTTACTGATCGCCGTACCGAAGG
GATACAGAGATTTTACAATGTCTGATTGGGAATTGGTTGGAATTCAAATTAAAGGACATACTAA
ACTGTGTAAATTTCGAATTAAAAGTATCGATGAAAGTTAACATTTATGTTGTGGGTGCCATTTT
GAAGTCATCATTTAATGTAATGCAAAAGTATTAGAGGCTTTTTTTTGATTTTGAATGTTTTTAA
AATTGATGATTTTTACAACTTCAAGTGCGTTGTATTCATAGAAGTACTCTGATTTTGCAATTGT
AAAAATGAATTTTTGTGGAAATTTTCATGATATTCTTGTGTTATATGGAACGAAAATAGTAGAC
ATTTTTGAAAACTAGATATTTCCAAG*TTCTTTT*ATTCAACAAAATCATAAAAATAAAAAAAAAT
ATTGCACTTCAAAATCACGAA

sltxB on NW_022197768.1:

AATCACATCCAGCACTTTCCATCTGAGTATTTGAAAAGAGTTTAATAGACCATAAATATGTACG
TAAAAATGATTTTACTGATTACATTCAGCCCATATGTCACAGAGAGAACACAGATTTAATTGAC
TTGCATTATATAAGTAAAAGATTTATCAGCAAT**ATGTTTTGGCAACTTATACTGCTTGCTTTCT**
TTGCAAGTGTTCGGGCAACTGAAGAAGAGCAAGCAGACGGTAAGATGTAGCTACAACTATTATA
AATTAATTTATATTTGAAACAAGTTTCTGTTTTTTTTTAAG**GAAACGGAACACCATATGAAGACG**
TGTGCGTCGGACTGATTGAATCAATAAGGTTTCAATATGAGTTTATAGAAGGGACCGGCAGGAA
AGATATTATATGGGTGAACAAGAAAACAATGAAACTCTTTTCATATGTGTCTATTATTTCTATT
ATTTGTTGATTATTTTGAATTATTTTTATTCGATTCTCCGCTAG**ATAAAATTGGATTCAGAAAT**
GCAAGAGAACTATACTAATAAGCAAGATTTTGCGTACGCACTAAATGAAGCATTCCTGAGAAAG
GCCCGAGTACGTTTGCTGACCAAAACATGTCGTACTGGTGATTTTGGATTTGGACAATTTGAAA
TATTGGACATAGGACAGCAGAAATAAAAATATTTATTTAAATTTAAAAACAGCCGTTTCGCAAT
CGGTTTTTATGGTCATTTGTAACAATATTCCGTTAATTGTAATTCGATTTATA*TTGTTT*AGAGA
AGAATTCTGAATGAATAAATGTATACACAATTAATAAtgccttaaaattaattcattggtgagt
aaatatatactaacattatcactacagcatagaacataaagaaattgtgaccaaaatctcacat
attttcgatgacacacaacctgaatttgcaatttgagtatcatgagtcaacaatgttcattttt
cattttcaatttaaatgattgtgtttcaaactaaagaaaataagtccaagtttttttttaaatt
catttgtgaattctttgaagcgacatttttttaaatatccaagcaagcgtttggtacctttttcc
aaaaaaattttcgttaaaattcaatcaaaattaactcatttatggaaatatttatttcatacat
ttttcacacttaattattacacatattgctgcctcaacatatcataagaaaagtccatgaaaat
tttctttttcgaaatttatttttatcaattctatagaacgtttccatttaaattagtttatggt
tgattagctatgacggcgttgcttatatatttgagtatttataagacttgacttattaagcact
ttcccacttaTACTTGAAGTGCAG**ATGACATCACAACTAT**GTAAGTCAGATATAATTTCGTTGC
TCGAAAATATATTGCTGGAAGTCCACCCTGTTTAAACGAATATTTTTTCAATTTTTTTTTTTTT
TTTCAAATGTATACTAG**CAAATAAGATTGATAGCAATTTGGCGGATATGTTTTTGCTCGTGATA**
CTATCGATTTACCTTTCGATTTCATCAGCTTATGTCAACGTAAGCCATGCAGTCTCTTTCCTTC
ATCCATATTTTGGAGGTAAGTTGCAACAATATAGCAATAAAGTCGAAATTACTATTTGAATTCA
ATAAGCTTAGATCTCACGATTATACACGCTTATAAAGATGTCATGTAATAATATCTCACATTTC
TATTGTTTCTGCCTTAG**AATATGCTGGAAATGATTATAAATATGAGAATTTAATGTCGAGATCG**

ATGCCAACGACTACACCACAGCCAAACATACCTGATTTTGATGAAGGAGATGATAGCTGTGTTG
GAAAAATAGAATCAATCCGATTTTTAACTGGACCGTTGCATGGACATATTCGTGATCACATAAC
TGTATTTAAATTGAATACCAAAAACTTTGAACATCATACGGATGTAAAAGATCTTCGAATTCCC
CTATATTTGTCATACAAAGCACAGTCTATTATAAGATTAGATATATCAAATTGTAAAAACAACG
AAATTTCAGTTAAGGGATTTACTATAGGTTTCATAGGCAATCCACCAGCTATAACAAATTTTCT
TTTATAACAAACACGCTTTATTTGTTCAA*TTTAAATT*CCAAATAAACTTTACCTAAAATATTAA
ATCTACTTTTGGAAGCAACGACA*TTTTT*AATTCAAACCAAATAATACATTTTCATCAGAAGGAC
TCTGATAATTACTGACCAGAGAAAACAAGGTT

M. destructor

Legend:

- Predicted exons are highlighted in blue and predicted introns are highlighted in yellow. Exon/intron boundaries for *M. destructor* are taken from Ensembl.
- Coding sequences are indicated in bold text. 5' and 3' UTRs are therefore designated by unbolded text highlighted in blue. The sequences for unannotated HGT candidates are highlighted in orange, with putative in-frame start and stop codons indicated in bold orange text.
- Poly(A) signals or cleavage sites are underlined. Upstream and downstream sequence elements are italicized.
- Initiator sequences are highlighted in white text when found within annotated mRNAs, or in blue text if found outside the gene boundaries.

rhs on AEGA01002600.1:

TTGATTATATGTGTCAGCATTATTTTAACCACCACAAAGATATGCACTATTAATCGAGAACAAA
GAGATTTGGAAAAGCAGTATTATGATTTTGTTGTGCGGCGTCTAAACATTTAAATATTAAATAA
ATAAATAAAATAAAAATTGGAAAAATTGGAAAGGAGAGTCTTCAGAAAGTGAAGAAATTATTTC
ACATAGTATGTTGAATTGATAAAGTTGTTATTGTTGTGCCTTAACTTTTTTTTTATTAATCCCT
TTTTATCTTAAATTTCGGTAACAGGTACAAGAATCCATTAGCAACCATTTTCA**ATGGCCGTAGA**
CAATGGATTTTTTTCGAATGCCAGTAATTTTCCTGCTCGCCACACGCACCACAGAGAAAAATGG
TTTAAAGCGTCGACAAGAAAAATTCAGTTATGATAATCGCAATCGATTAATTAGCTACAATGCA
TCGGGCGATAGCCTTCCGGTGGATTCGTATGGAAATTTGATGACTTCACAAACATATCGATATG
ATGCACTGAATAATATCATTTCTATAAGAACAACATTGTTTGATAATTCTGTGGATAATGTTAC
GTATCATTATCTCAATCCCGATGATCCAACACAGTTGACGAAAGTAACACATACACACAAGAAA
TATCCCGAAACCATTATGTTGAGTTATGACACTGAAGGCAGGCTGACTTGCGATGAAGCAGGAA
GATTACTTTCTTATGATGTTTTGGGACGATTGATCAGCGTCAATGGAAACAACGATCGTTCAAG
AATGTACAGCTATGATGCACTCAATCGTTTAATCGCCAAAAAAACCAGTAAAAATAATGAGATC
CAAGAATTGTATTATCGTGGTTCGGAATTGGTGAATGAAGTGATAAATTCACAGAAAAAAGAAA
AACGTTTCATCAAAAATGGCCACGAATGTCTGGGCGTAAGTGACATCAATGGTCTTACAATAAC
GGTTGGCGATAAAAACAACAGCCTTTTGTTGTCCAAAAATGTGAATTTCGGCAGTGAAGATATT
CAGTGCCATGTTTGGTCACCCTATGGCAGCAGTACCTCGACTGATAATCGTCTTTTAGGTTTAA
ACGGTGAACGTTTTGATTTAGCCAGTGGTACATACCATCTTGGAAATGGTTATCGTGCATATAA
CCCAGTTTTGATGCGTTTTAACTGCCCGGACAATTTAAGTCCATTTAGTGCCGGTGGAATAAAT
CCATATGCTTATTGTGCAGGAGATCCAGTAAATCATATTGATCCATCTGGCCATTTCAGTTGGA
TAGCTATGACTGGAATAACTCTGAATATAGTTGGACTCGCCCTATCTGTTTTCACGGCTGGAGC

GTCTATTGCGGCTGCAGGTAGTGTGATGGCAGCTATAAGTTCGGCTTCGGCTTCCGGATTGATC
ATCGGTTCATTGAACGTAGCTTCAGATATAACGGGGATAGCAGGTGGATCAATTGCAATTTTTA
ATCCGGAAGCGTCGTCAGCATTGGCTTGGATGTCGTTGGCATTAGGATTTTATAGCATGCGAAG
ACCCATCGGAGAATTCAAATGGCTTAAGACTGGTATCGACGATTCCATATTTGCAGAACATATT
GTCGATGATTTACCCAAAAACAGATTCACTGCAGTCACAAGTTGATCCACGTACTGGCCAATTT
ATGTTGAATTTTCCAGTTGCTGAGTTAATTGGAAATAATCAACTTGGTCCTGTATTATCGCTGT
CTTTGAAGTATTCGCCGTTGAATGGAGAAAATGAAGGATTTGGAATCGGATTTTCGATTGGACT
TACGCGATTCAACAGTCGAACCCATTCACTAAATCTTAGTAATGGCGAACAATATCGTGTCGGC
ATAGGGGCATATTGAAACTGGCAAGTGGTACGAAACTGCTTCATGCGTTCGAAATTCATTCGGA
GAAATCATAGCAGAAACTGCACAAGATTGGTTGACAAATAGCTCAGACAGTGAACAGTTGTTTT
CAATAAAATCAGAAATGATTGATGGTGGATGGGAAGTAATCACTAAAAATATCAGCAATAAATC
ATTTTCATTAAAATCAAATATAATTCATGATGGATGGGGAATGAAGAAAGAAATTAATTTCTCC
AATGGCGTGAAACGTCTTCAGAAAATCGATCCTATTAATTTGACTGAATCGGTTTATAATCGTG
AAATGTCGGGATCCAATCCATTGATTTGCGCTGAAATGTTCATCGAAAAGGATAAAACAAGCGA
GTTTCCTGTCAGAATGATCATGAACGACACAATGGGTAACGAATACAATCATTGTACCTATAGC
TGGAACGGACTGGGCCAATTGCTCGAAGAACAAGATGAACTACAAGCTGTCACAAAAAGAACTT
ATGATCCATATGACCGAGTATTGACACAAATATTGCCTGATGGAACTATTTTAAAACAAACTTA
TGCACCACATTCAACCGAAAATAAGATTGCTTCGATTAGTGTGACTGGTATGAATGGCGAAGGA
AATGTAAAGACTTGGTTGTTGGGTACACAAAAGTTTGATAGCTTAGGAAGATTGACGGAGTGTG
CTAGTGGTGGCCGCACTACAGTGTATAGCTATTCAGATGCTTCGGCTGTTCCATCATTAGTTAC
TTTGCCATCCGGTAAAACTGTGACATACACTTACATCCCAGAATTGGGCAATTCAATCAGAAGT
ATGAAAGCGGATGGTATCAGTCAAGATTTTCGCTATGCCCCAAGGTCAGGAAAATTATTAATGG
CGCGAGAAGCCGAATCGAAAGTCGAAAAAAATTGGTCGTCAAATGGTCAGCTGAAATATGAGGT
ATTCTCGCTTAATGAAGATGCTCGTCGAGCCGAATATAAATACACATTGAATGGAACACTAGTT
ACGTATACTGATGTAGCAGGCAAAAAAATGCAATACATTATGGATGAGCATGGTCGAACCATTC
AAATAATCGATGATAGCTTAACGATTGATTTGTCATATGATGCTTTGGGCCGTTTAACAGCACA
GAAAATCCAGAGCTGTGGATCACCTACTTTTGTGAAAACTGCATTAAACTACGACATTTTCGGC
CGGGAGATCGAGCGCCACATTATTGATAATAAGGATATGACCTTGATTTTATCGCAGACGTGGC
AGAAAAATGGTTCAGATAAAGTGCTTCATCAAAAGCTTAAAAATTTTCGCTTTTGTTATACAAA
TGGCCATAATGAAGATGAAGGCTACACTATATTTTGGAAAGAGGGTAAAATAGAAAAACTATCG
AAAACCGAAGATGATGTCACATTTGTCACCTCGTTCATTATGTCACCATTGGGCCGGCACATGA
AATTATCTTGGGACTGGAGTGGTCAATATGCACGACTAATAAAAATCGAAGACGAATTCAAAAT
TTTATGCAAAATAAATTACAATACAAACGTACAAGTTGAAATCTGGCCGAATACATCAGATGCA
TATAAATTGAATTTTGAACTCATCAATGATGGTCAATTGGATACAATAGCCCGTAAAGTATCAG
ATTCGAACACTCTTAATTGGAATTTTATTTACGAGGATGTTGGTCACATGAGTAACCGATTGAC
ATTGACTGGTGTTAATTATCCTACTCACATGCAAGATACCGTTGAATATAAAACACAAGATGGA
TTACCATTTCCAAACAAATCTGGTCGTCACTTAAAACTTCCTTGTGTTCAGACATACACACGCA
ACGTGGGATTTGGTCAACCGGAAACTATTTGTTTCTACGAATACACTCGAAATAATTTTCTGGG
ATATGATGGTGATTTCGGTGACTGGTCTGCTGACAGCGATTACCTTTATACAACTCTAACAGAT
TACACATATGGATCAAAAGAGAAGTCAGTGTGTGGTGACATCTCCATTGTTACTGAGCGAACAT
ACAACAACTATCATTTACTAATCGCTGAAGAGATCAACAGACAAAACCATATTCATCGTACAGA
ATACAGTTATTATGCCGTGAAAGATTGTTTCATTGATGGCCAACCAGCTCAATTCCAATTGCCC
AAGGTGAAAAAAGAGATCATGCGAGATCCAACGGGTAATGTTCGTACGTTAGTAATGCATAGTG
AATTCGATGAAAATGGCAATCCAACGAAAGAAGTACATCCAAATGAAACGGTTACGATAACAAC
TTGGTATAAAGCAGAAGGTGAAAATGGATGTCCAGCCGAACCAAATGGCTTTGTTCGTTTCATG
AAGGAACAACGAAATATTCCCCGTCATATTACTGCATTTGAACCGGAATATTCTACAAAGTACC
GTTACACTAAGCTCAGTGACACCAGATACGTAGTACAAGAAAGTAAAACATCTTTTTGTGATGA

TTTCACACTTAGTGAGCGGCGTTGGGCATATGATGAGAATAAAAGTAATGAATTGGGCCGGATT
GTGTCGATTAGTGATACTGTGTACGATTTGTCTGATAAATCAAAGTGTTACACTTCCACACAAC
GTTTTACGACAACAGTTATAGACAATCAAATGACACAGAAAATCATTTTCACTGGACATGATGA
TTTGCAACAAACATCAACGCGATGTCAATCTGTCTTTAATAGTCGATTGTTTAGTGAAATCTCT
TCACTTGGACTAGAAACACAGTATAGTTATGATCAATTGGGAAGGTTGGTATACCGCAAGCTAT
GTCCAAATTCGGATTATGAAAACCTCACCACATGGGAATATGATATCAATGATGAGGGCTTACA
TTCAATCAAAACGGATGCTTCGGGAAATAAAGAGAAGACATGTTTTGATGGTACTGGACGCGCT
ATAAGTTGGGAAAATTTTGAGTATTCTATCAAAAGAGTTGTTTTAAAATAGAATACAATAATTA
TTATTCGTATTATTACTTACAG**ATCTTTCTATGGAAAAAAAGGGCTTGAAGTATATGGAGGAGC**
GTGGAAGCCACCAAAGTGGACAATTCCGCATAATAAATTAGATGGTTTGCAAGTAGGACAGGAA
GCGAATGCTTTTCGTGTTGAGTCTAAATTGTTTGCCGTTCGGAAAGATTCTATATTTAATGAAA
ATGGCACAAGAATTGCTGAGAAAAGAAACTGGATAAAGAGCAGAAGTTCCAACACTTTTGAAAA
TGGAATTGATTCACAAAAACCAGCCTTCGCCCGGATGAATCGTATGGCCAATATGCGAAATAGA
AACGTAACGAAACGTATTGAAAAATCACGACAACGAACACCACTCATATATAGCTTTGGCAGGA
ATGACCCCATCTGGTATCTTCTTGACTAAATGAAATCCTGAGTTATACACATATTTTGACTATT
ACATATGATCTTACTGTAACAACTAACATTAAAAATGATTTATAAATAAATAATCATAACAAAT
ATGCTTGATCAATCATCAT

aip56 on AEGA01003780.1:

ATTCAGTCTTTGACACGAACCGTATCATTACGTTTTTGGTGGATGTGGAAATGAGTCTAAAAAT
TTTTGTTTTGTGCTTTTGTTGCATTACAATCGCCAATAGTGAACCATTCAGGGCA**ATG**ATTGAA
TGTTTAAGACTTTGTACTCAACCCCCAATTGGACCGCCTGTGATCCATTATAGCAGTTTGTCGC
TTCGCTATGGTGAAGATACGCGCATCCCGCCTCAAATCCATCATATTATACCTGCATCAAGACT
TAGAAGTTTCTTCAACTTTGCTTTAGGTGATAGGAGAGCAACCGTAGCCCGGGATTTCATTGAA
TTTTTAAAGGAGTTAGCAGAATTTGCAATGTATAGCTATTTGCCAAGTCATGTGCCAGCCGATT
TATGCGATTTTATTAATGATATAGACAATCCAAATAGAAATGTTTTTCACAATTGGTCGGAATT
GACGCCCGAACGACGAACGGAAATTTTAAATGCCCTAACTGGCTATGGACGCATTGTAGTTGAT
ATGTTCCAAATGATGCCATTCAACTTGGCCATTGGCCCAATGGAAAGATCTGACGACCCTGGTG
GAGCATTCGATCAACCCATGCAGTATATAGTTGAATCAGAACATTACAACGCGTTGCAAAGCAT
AAATAGATATATGGGTGGTGCTAATAGCCATGCAGATCTTCATTATGTTCAAGAAACAATAAGG
TTATTAAGAAAAATGTTGGCGAATAATATAAGACGAGAACCGTACAGATATAATGAGGACGATT
GGGACCGCAATAAATACACCAAAAAGTTTAGTGTTAAAAAACAATCGAGACCTCGCCGCCAAAC
GGCTGAACTGTTCCCTGATATTGCATATTCACTGTTTGGTTTTTTGCCGGCGAGAGTGCGCAGG
GAATCACAATCGTGTCAGGACAGAACCCGTTATTCTGAAATAACTATCAAAAGCGACGAATCCT
ATTTTGAGAACGATCCTATGTGTCTAAATTCGTTTGCCAATTATAACCACGCAAGGAAAGTAGT
TTTATTTATTATCAAC**TAA**TTTAGCAAAGTCTCTAATGTTGACATCGAATGAATTCCGGTTTTT
TTAAAAATACTACTACATATTACATAAATAATACTAACGACGAAATATTGTATGATCGGAATTT
TTATAGCTTTTCTCATTAATTTGATCCATAGGTGTATAATCTGTACCCTTTCTATTACAGCCAG
ATTTCGGCGGTGTGA*TTTGATTTT*AAATAAATAATTTTTTTTTTTTTAAATAAACATATA*TTTT*
*TTTT*ATTGA

Table S1. CdtB sequence QDF82160 from *Scaptomyza* nr. *flava* was used as a BLASTP query to the NCBI GenBank: Eukarya (taxid: 2759) database on 9/28/2020. These results show that *Co. nasturtii* was a hit (**Table S1a).** Another search with this newly discovered sequence, XP_031639861.1, as query shows two that there are two possible *cdtB* copies in the *Co. nasturtii* genome (**Table S1b**).

Description	Max Score	Total Score	Query Cover	E value	Per. Ident	Accession
cytolethal distending toxin B [Scaptomyza nr. flava (dark) KIV-2019]	296	296	100%	3e-102	100.00%	QDF82160.1
cytolethal distending toxin B [Scaptomyza nr. nigrita KIV-2019]	235	235	100%	1e-77	80.56%	QDF82161.1
cytolethal distending toxin B [Scaptomyza pallida]	160	160	100%	3e-48	57.72%	QDF82159.1
cytolethal distending toxin B [Drosophila primaeva]	135	135	88%	2e-36	53.03%	QDF82163.1
uncharacterized protein LOC116351853 [Contarinia nasturtii]	63.5	63.5	99%	2e-09	31.79%	XP_031639861.1
uncharacterized protein LOC116654561 [Drosophila ananassae]	60.8	60.8	88%	3e-08	29.10%	XP_032305712.1
PREDICTED: uncharacterized protein LOC108127405 [Drosophila bipectinata]	60.5	60.5	90%	4e-08	30.00%	XP_017099943.1
uncharacterized protein LOC111028693 [Myzus persicae]	53.1	53.1	88%	1e-05	25.71%	XP_022163116.1

Table S1a.

Description	Max Score	Total Score	Query Cover	E value	Per. Ident	Accession
cytolethal distending toxin B [Scaptomyza nr. flava (dark) KIV-2019]	296	296	100%	3e-102	100.00%	QDF82160.1
cytolethal distending toxin B [Scaptomyza nr. nigrita KIV-2019]	235	235	100%	1e-77	80.56%	QDF82161.1
cytolethal distending toxin B [Scaptomyza pallida]	160	160	100%	3e-48	57.72%	QDF82159.1
cytolethal distending toxin B [Drosophila primaeva]	135	135	88%	2e-36	53.03%	QDF82163.1
uncharacterized protein LOC116351853 [Contarinia nasturtii]	63.5	63.5	99%	2e-09	31.79%	XP_031639861.1
uncharacterized protein LOC116654561 [Drosophila ananassae]	60.8	60.8	88%	3e-08	29.10%	XP_032305712.1
PREDICTED: uncharacterized protein LOC108127405 [Drosophila bipectinata]	60.5	60.5	90%	4e-08	30.00%	XP_017099943.1
uncharacterized protein LOC111028693 [Myzus persicae]	53.1	53.1	88%	1e-05	25.71%	XP_022163116.1

Table S1b.

Table S2. Genomic and transcriptomic resources utilized in the text. **Table S2a** shows APSE genomes whose proteomes were used as queries against cecidomyiid genomes. **Table S2b** includes cecidomyiid genome and transcriptome information.

Table S2a. APSE genomic resources.

**APSE Type**	***Hamiltonella* strain**	**Insect Host**	**Toxin**	**Assembly ID**	**Fragment ID**
APSE-1	NA	*Acyrthosiphon pisum*	Shiga-like	ENA: AF157835.1; GCF_000837745.1	
APSE-2	5AT; NY26; 82B; ZA17; WA4	*Acyrthosiphon pisum*	CdtB	GCA_000882435.1; ENA EU794049.1	
APSE-3	A1A; A2F; AS3; AS5; R7; H76	*Acyrthosiphon pisum; Aphis fabae*	YD-repeat	LR794150; GCA_902859955.1	EU794053.1; EU794057.1
APSE-4	5ATac	*Aphis craccivora*	Shiga-like		EU794051; EU794056.1
APSE-5	NA	*Uroleucon rudbeckiae*	Shiga-like		EU794050; EU794055.1
APSE-6	N4; H402	*Chaitophorus sp.; Aphis fabae*	CdtB		EU794054.1
APSE-7	NA		CdtB	ENA: LR794147; GCA_902859665	EU794052
APSE-8	3293	*Cinara watanabei*		ENA: LR794148	

Table S2b. Cecidomyiidae genomic resources.

**Species**	**Taxonomy**	**Type**	**Notes**	**GenBank/SRA ID**
Ca. subobsoleta	Cecidomyiidae: Lestremiinae: Catotrichini: Catotricha	Genome		GCA_011634745.1
Ca. subobsoleta	Cecidomyiidae: Lestremiinae: Catotrichini: Catotricha	gDNA reads		SRX7007124
Co. nasturtii	Cecidomyiidae: Cecidomyiinae: Cecidomyiini: Contarinia	Genome		GCA_009176525.2
Co. nasturtii	Cecidomyiidae: Cecidomyiinae: Cecidomyiini: Contarinia	gDNA reads		SRX6846370
Co. nasturtii	Cecidomyiidae: Cecidomyiinae: Cecidomyiini: Contarinia	transcriptome	L1	SAMN12767267
M. destructor	Cecidomyiidae: Cecidomyiinae: Oligotrophini: Mayetiolia	Genome		GCA_000149185.1
M. destructor	Cecidomyiidae: Cecidomyiinae: Oligotrophini: Mayetiolia	gDNA reads		SRX020192
M. destructor	Cecidomyiidae: Cecidomyiinae: Oligotrophini: Mayetiolia	Transcriptome	male L1	SRX545609
Si. mosellana	Cecidomyiidae: Cecidomyiinae: Clinodiplosini: Sitodiplosis	Genome	Single pupa	GCA_009176505.1
Si. mosellana	Cecidomyiidae: Cecidomyiinae: Clinodiplosini: Sitodiplosis	gDNA reads	Single pupa	SRX6820651
Si. mosellana	Cecidomyiidae: Cecidomyiinae: Clinodiplosini: Sitodiplosis	Transcriptome	Non-diapause larvae	SRX312060

Table S3. Patterns of micro-synteny show that *sltxB* was likely transferred in Cecidomyiidae prior to the divergence of *Co. nasturtii* and *Si. mosellana*, but fragmented genomes make the relative timing of the other horizontally transferred genes difficult to determine. Text highlighted in yellow is the locus (i.e. protein GenBank ID) of interest. "Position relative to HTG" indicates the number of genes upstream or downstream of the gene of interest. For example, -5 would indicate the gene is five genes upstream of the HTG. Cells highlighted in blue indicate genes are on the same scaffold (and, presumably, syntenic) with the HTG in the considered species.

	**Species**	**ID type**	**-5**	**-4**	**-3**	**-2**	**-1**	**0 (HTG)**	**1**	**2**	**3**	**4**	**5**
aip56													
**Query**	***Co. nasturtii***	**Query ID**	XP_031641173.1	XP_031616390.1	XP_031641173.1	XP_031640490.1	XP_031640489.1	XP_031641113.1	XP_031616417.1	XP_031640808.1	ref|XP_031619577.1	XP_031640287.1	XP_031640285.1
**DB**	***M. destructor***	**Scaffold**	GL501538.1	GL501545.1	GL501538.1	AEGA01028632.1	GL501538.1	GL501532	GL501450.1	GL501523.1	GL502152.1	GL502152.1	GL502152.1
**DB**	***Si. mosellana***	**Scaffold**	VUAH01006196.1	VUAH01006196.1	VUAH01006196.1	VUAH01006196.1	VUAH01006196.1	NA	VUAH01000002.1	VUAH01006225.1	VUAH01006165.1	VUAH01006165.1	VUAH01006165.1
aip56													
**Query**	***Co. nasturtii***	**Query**	XP_031636924.1	XP_031636923.1	XP_031636931.1	XP_031636947.1	XP_031636945.1	XP_031636937	XP_031636922.1	XP_031636912.1	XP_031636910.1	XP_031636972.1	XP_031636906.1
**DB**	***M. destructor***	**Scaffold**	NA	AEGA01015109.1	AEGA01031561.1	AEGA01015432	AEGA01025736.1	AEGA01034427.1 [<1 kb]	NA	AEGA01025736.1	AEGA01025736.1	AEGA01008845.1	AEGA01020694.1
**DB**	***Si. mosellana***	**Scaffold**	NA	VUAH01000002.1	VUAH01006169.1	VUAH01006169.1	VUAH01006169.1	VUAH01001532.1 [< 5kb]	NA	VUAH01006169	VUAH01006169.1	VUAH01000002.1	VUAH01005947.1
aip56													
**Query**	***M. destructor***	**Query**	Mdes005436	Mdes005438	Mdes005440	Mdes005441	Mdes005442	GL501532 [1200694-1201296]	Mdes018772	Mdes005446	Mdes005448	Mdes005449	Mdes005451
**DB**	***Si. mosellana***	Scaffold	NA	VUAH01001550.1	VUAH01005279.1	VUAH01000010.1	NA	VUAH01006225.1	VUAH01000037.1	VUAH01006149.1	VUAH01000037.1	VUAH01006149.1	VUAH01006196.1
sltxB													
**Query**	***Co. nasturtii***	**Query**	XP_031620142.1	XP_031620140.1	XP_031619571.1	XP_031619573.1	XP_031619575.1	XP_031619578.1	XP_031619577.1	XP_031619799.1	XP_031620054.1	XP_031619946.1	XP_031619607.1
**DB**	***Si. mosellana***	**Scaffold**	VUAH01006166.1	VUAH01006166.1	VUAH01006166.1	VUAH01006166.1	VUAH01006166.1	VUAH01006166.1	VUAH01000029.1	VUAH01006166.1	VUAH01006225.1	VUAH01006225.1	VUAH01006225.1
lysozyme													
**Query**	***Co. nasturtii***	**Query ID**	XP_031638452.1	XP_031638329.1	XP_031638327.1	XP_031638384.1	XP_031638801.1	XP_031638744.1	XP_031638337.1	XP_031638715.1	XP_031638692.1	XP_031638673.1	XP_031638439.1

**DB**	***M. destructor***	**Scaffold**	GL501497.1 [69213-70070]	GL501436.1	AEGA01033347.1	GL501523.1	GL501532.1	GL501497.1 [230410-230853]	GL502903.1	GL502903.1	GL502903.1	GL502903.1	GL502903.1
**DB**	***Si. mosellana***	**Scaffold**	VUAH01000001.1	VUAH01000003.1	VUAH01000001.1	VUAH01000001.1	VUAH01003258.1	NA	VUAH01000001.1	VUAH01000001.1	VUAH01000001.1	VUAH01000001.1	VUAH01000001.1
rhs													
**Query**	***M. destructive***	**Query**	Mdes011029	Mdes011030	Mdes011031	Mdes011032	Mdes011033-RA	Mdes011034	Mdes011036	Mdes-011038	Mdes011041	Mdes011042	Mdes011045
**DB**	***Si. mosellana***	**Scaffold**	VUAH01000041.1	VUAH01000041.1	VUAH01000003.1	VUAH01000005.1	VUAH01000005.1	VUAH01005136 [<13 kb]	VUAH01006196.1	VUAH01000005.1	VUAH01000005.1	NA	NA
**DB**	***Co. nasturtii***	**Scaffold**	NW_022203985.1	NW_022198578.1	NW_022198383.1	NW_022197885.1	NW_022198040.1	NA	NW_022197981.1	NW_022198574.1	NW_022198574.1	NW_022198340.1	NW_022198038.1

Table S4. Approximately Unbiased (AU) test results comparing different topologies. Daggers indicate that the only insect tips are cecids, while asterisks indicate statistical significance. In this table, "APSE" refers to both APSE phages and *H. defensa*. Details of forced monophyly are shown in **Table S10**.

**AIP56**	**AU**
Default	0.428
Cecid-Mono	0.753
Insect-Mono	0.148
Cecid/APSE-Mono	3e-05*
**CdtB**	**AU**
Default	0.511
Cecid-Mono	0.682
Insect-Mono	0.306
Cecid/APSE-Mono	0.299
**Lysozyme**	**AU**
Default	0.492
Cecid-Mono	0.520
Insect-Mono	0.445
Cecid/APSE-Mono	1e-004*
**RHS**	**AU**
Default	0.791
Cecid-Mono†	0.208
Cecid/APSE-Mono	4e-004*
**SltxB**	**AU**
Default	0.383
Cecid-Mono†	0.592
Cecid/APSE-Mono	0.515
Cecid/Proteobacteria-Mono	0.003*

Table S5. Phyre2 analyses of proteins encoded by horizontally transferred genes can provide clues as to the function of these proteins, even in eukaryotic genomes. **Table S5a-e** are Phyre2 analyses for, respectively: AIP56, CdtB, Lysozyme, RHS, and SltxB.

Table S5a. Phyre2 analyses of AIP56 sequences.

Query Sequence	Template	3D Model	Confidence	% id/coverage	Template Information
Hamiltonella defensa (WP_1000096555)	c6nobA	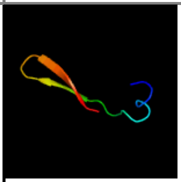	65.4	43/9	PDB header:hydrolase<br>Chain: A: PDB Molecule:beta-fructofuranosidase;<br>PDBTitle: structure of glycoside hydrolase family 32 from *Bifidobacterium adolescentis*
Photobacterium damselae (WP_012954632)	c4lgjA	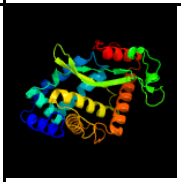	100.0	40/50	PDB header:hydrolase<br>Chain: A: PDB Molecule:uncharacterized protein;<br>PDBTitle: crystal structure and mechanism of a type iii secretion protease
Co. nasturtii (XP_031641113.1)	c2k19A	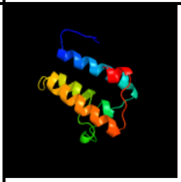	72.2	19/22	**PDB header**:antimicrobial protein<br>Chain: A: PDB Molecule:putative piscicolin 126 immunity protein;<br>PDBTitle: nmr solution structure of pisi
Bactrocera dorsalis (XP_014102626)	c2zx3B	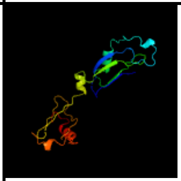	96.7	21/31	PDB header:immune system, sugar binding protein<br>Chain: B: PDB Molecule:csl3;<br>PDBTitle: rhamnose-binding lectin csl3
Arsenophonus nasoniae (WP_051297127)	c4jgjA	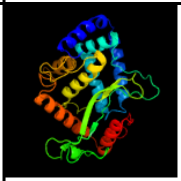	100	32/46	PDB header:hydrolase<br>Chain: A: PDB Molecule:uncharacterized protein;<br>PDBTitle: crystal structure and mechanism of a type iii secretion protease
M. destructor (GL501532)	c5lnkW	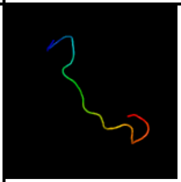	47.3	28/8	PDB header:oxidoreductase<br>Chain: W: PDB Molecule:mitochondrial complex i, sgdh subunit;<br>PDBTitle: entire ovine respiratory complex i
Hamiltonella defensa (WP_10009655)	c6nobA	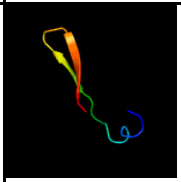	65.4	43/9	PDB header:hydrolase<br>Chain: A: PDB Molecule:beta-fructofuranosidase;<br>PDBTitle: structure of glycoside hydrolase family 32 from *Bifidobacterium adolescentis*

Table S5b. Phyre2 analyses of CdtB sequences.

**Query Sequence**	**Template**	**3D Model**	**Confidence**	**% id/coverage**	**Template Information**
Bacteriophage APSE-2 (AGX01517.1)	d2f1na1	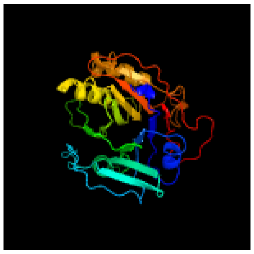	100.0	36/85	Fold:DNase I-like<br>Superfamily:DNase I-like<br>Family:DNase I-like
Co. nasturtii (XP_031641203.1)	d2f1na1	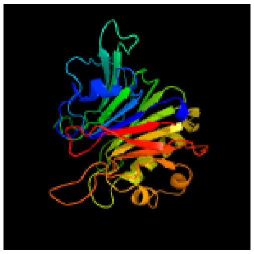	100.0	29/91	Fold: DNase I-like<br>Superfamily: DNase I-like<br>Family: DNase I-like
Co. nasturtii (XP_031639861.1)	c4k6lF_	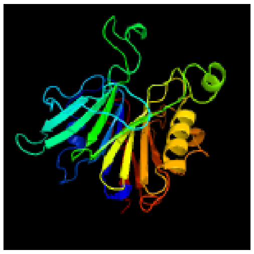	100.0	28/95	PDB header: toxin<br>Chain: F: PDB<br>Molecule:cytolethal distending toxin subunit b homolog;<br>PDBTitle: structure of typhoid toxin

Table S5c. Phyre2 analyses of lysozyme sequences.

Query Sequence	Template	3D Model	Confidence	% id / coverage	Template Information
Co. nasturtii XP_031638744	c6et6a	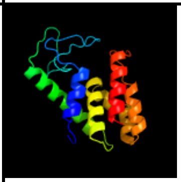	100	36 / 99	PDB header:hydrolase Chain: B: PDB Molecule:lysozyme; PDBTitle: muramidase domain of spmx from *Asticaccaulis excentricus*
M. destructor (GL501497 [230410-230853]	c6et6a	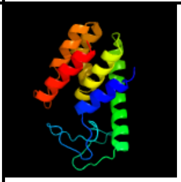	100	42 / 98	PDB header: antimicrobial protein Chain: A: PDB Molecule:lysozyme; PDBTitle: crystal structure of muramidase from *Acinetobacter baumanni*i ab 5075uw2 prophage
Coprinopsis cinerea okayama7 #130 (XP_001840847)	d1xtja	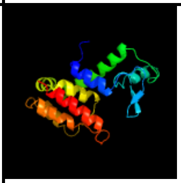	100	26 / 21	Fold:Lysozyme-like Superfamily:Lysozyme-like Family:Phage lysozyme

Table S5d. Phyre2 analyses of RHS sequences.

Query Sequence	Template	3D Model	Confidence	% id/coverage	Template Information
Xenorhabdus vietnamensis (WP_086110724.1)	c4o9xA_	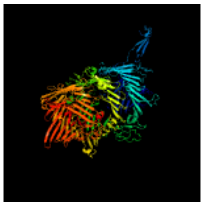	100.0	17/80	PDB header:toxin Chain: A: PDB Molecule:tcdb2, tccc3; PDBTitle: crystal structure of tcdb2-tccc3
M. destructor (GL501425[246422,251816])	c4o9xA_	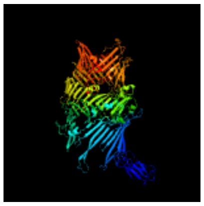	100.0	17/89	PDB header:toxin Chain: A: PDB Molecule:tcdb2, tccc3; PDBTitle: crystal structure of tcdb2-tccc3
Si. mosellana (VUAH01006948[8,4950])	c4o9xA_	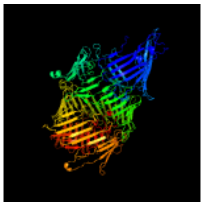	100.0	16/74	PDB header:toxin Chain: A: PDB Molecule:tcdb2, tccc3; PDBTitle: crystal structure of tcdb2-tccc3

Table S5e. Phyre2 analyses of SltxB sequences.

Query Sequence	Template	3D Model	Confidence	% id / coverage	Template Information
Co. nasturtii (XP_03169577.1)	c5mgfC	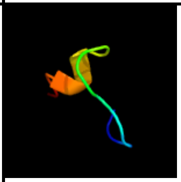	42.8	69/14	PDB header:splicing<br>Chain: C: PDB Molecule:snw domain-containing protein 1;<br>PDBTitle: cryo-em structure of a human spliceosome activated for step 2 of2 splicing (c* complex)
Co. nasturtii (XP_031619578.1)	c4n6cB	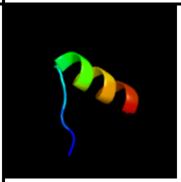	20.2	22/19	PDB header:structural genomics, unknown function<br>Chain: B: PDB Molecule:uncharacterized protein;<br>PDBTitle: crystal structure of the b1rzq2 protein from *Streptococcus pneumoniae*. northeast structural genomics consortium (nesg) target spr36
Si. mosellana (VUAH01006166)	c3vgxD	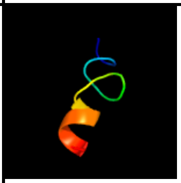	28.3	67/25	PDB header: membrane protein<br>Chain: D<br>PDB Molecule: envelope glycoprotein gp160;<br>PDBTitle: structure of gp41 t21/cp621-652
Si. mosellana (VUAH1000060.1 [425037-425252])	d2bosa	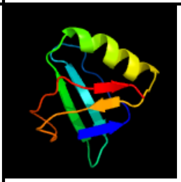	92.4	31/97	Fold:OB-fold<br>Superfamily:Bacterial enterotoxins<br>Family:Bacterial AB5 toxins, B-subunits
Si. mosellana (VUAH1000060.1 [423461-423673])	d2ogga2	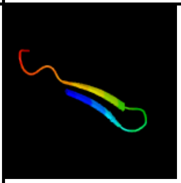	42.1	38/21	Fold:Polo-box domain<br>Superfamily:Polo-box domain<br>Family:Polo-box duplicated region
Si. mosellana (VUAH1000016.1 [901146-901313])	c3pl0B	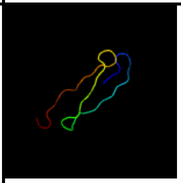	27.0	23/73	PDB header:biosynthetic protein<br>Chain: B: PDB Molecule:uncharacterized protein;<br>PDBTitle: crystal structure of a bsma homolog (mpe_a2762) from *Methylobium petroleophilum* pm1 at 1.91 a resolution
Si. mosellana (VUAH1000016.1 [899256-899522])	d1r4pb	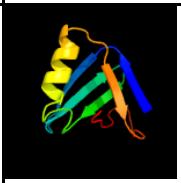	37.7	30/78	Fold:OB-fold<br>Superfamily:Bacterial enterotoxins<br>Family:Bacterial AB5 toxins, B-subunits

Si. mosellana (VUAH1000016.1 [900260-900502])	d2bosa	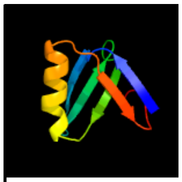	78.5	31/81	Fold:OB-fold Superfamily:Bacterial enterotoxins Family:Bacterial AB5 toxins, B-subunits
Si. mosellana (VUAH1000016.1 [896355-896597])	d2bosa	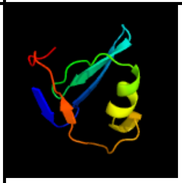	92.0	27/71	Fold:OB-fold Superfamily:Bacterial enterotoxins Family:Bacterial AB5 toxins, B-subunits
APSE-5 (ACJ10077.1)	d1c4ga	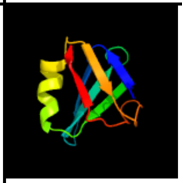	84.3	30/73	Fold:OB-fold Superfamily:Bacterial enterotoxins Family:Bacterial AB5 toxins, B-subunits
Bacterium associated with poplar plant (RYX79668.1)	d1c4ga	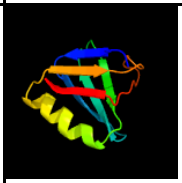	96.5	30/85	Fold:OB-fold Superfamily:Bacterial enterotoxins Family:Bacterial AB5 toxins, B-subunits

Table S6a. Primers, reaction details, and gel images for amplification of horizontally transferred genes in the *Co. nasturtii* (**Table S6a**) and *M. destructor* (**Table S6b**) nuclear genomes.

Table S6a. Primers, reaction details, and gel images for amplification of horizontally transferred genes in the *Co. nasturtii* nuclear genome. Invitrogen 1kb Plus Ladder was used as the marker.

**Primers (5'->3')**						
**F**	**R**	**Amplicon**	**Ta (˚C)**	**Extn. time (min:sec)**	**Size (bp)**	**Gel Image**
TCCGAAGACATGACAGTGCC	TTCAATCAGTCCGACGCACA	*aip56* copy on NW_022197544.1<br><br>(LOC116352557) and nearest eukaryotic gene (LOC116352216)	57	1:40	1604	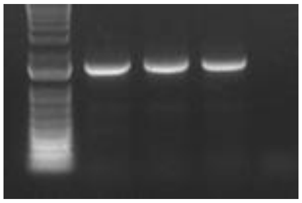
TGAATCCACGGCGAAGGAAA	ACCCACTAACGCAACCGAAT	*aip56* copy on NW_022200251.1 (LOC116349575) and nearest eukaryotic gene (LOC116349581)	57	1:40	1433	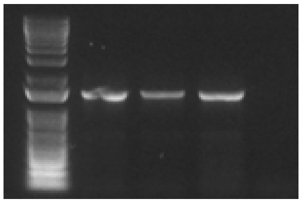
TCCATGATTGTCACGTGAAACA	TGCAAGGGAAATTAAAACGATCAGT	*cdtB* copy on short scaffold (LOC116351853)	52	1:10	1000	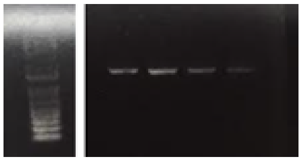
GGCGATTTCAACACAGAGCC	CCCCGAAATGCCTCTACCAT	*cdtB* copy on long scaffold (LOC116352617) and nearest eukaryotic gene (LOC116352014)	52	1:10	908	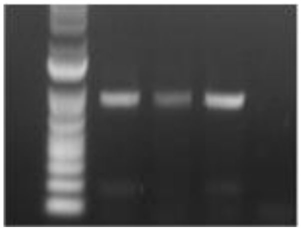

TCACCATTGTCCGTGCTCTC	CAAAGCGGGATCGTGCATTT	*lysozyme* copy 1 on NW_022201606.1 (LOC116350899) and nearest eukaryotic gene (LOC116350655)	57	1:15	1118	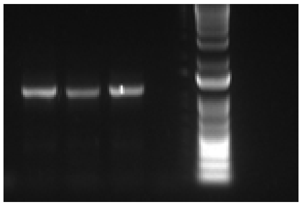
GATGCAACGTTACCACAGCC	ACAACGTGCATTTCGGAAGC	*lysozyme* copy 2 (NW_022201606.1: 2605394-2605828) and lysozyme copy 3 on NW_022201606.1(LOC116350797)	57	1:30	1508	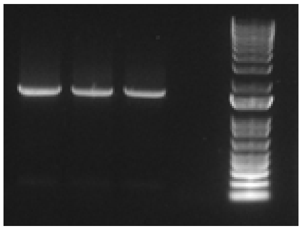
CCAATGTCACTGCAATCGCC	ACATCCGAAGCCTCATCGTC	*lysozyme* copy 3 on NW_022201606.1 (LOC116350797) and nearest eukaryotic gene (LOC116350628)	57	2:15	2166	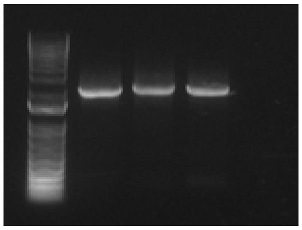
CCTACGAAGGGCGCTAACTG	TTCAATCAGTCCGACGCACA	*sltxB* copy 1 on NW_022197768.1 (LOC116338454) and nearest eukaryotic gene (LOC116338452)	57	1:15	1054	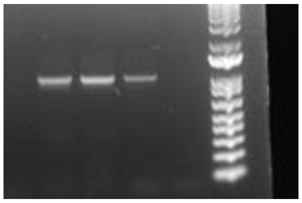
AGCATTCCTGAGAAAGGCCC	GGTGTAGTCGTTGGCATCGA	*sltxB* copy 2 on NW_022197768.1<br><br>(LOC116338453)<br><br>and *sltxB* copy 1 (LOC116338454)	57	1:30	1244	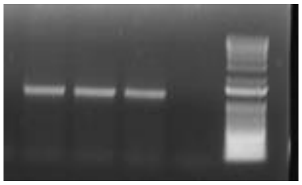

Table S6b. Primers, reaction details, and gel images for amplification of horizontally transferred genes in the *M. destructor* nuclear genome. NEB 100bp DNA Ladder was used as the marker.

**Primers (5’->3’)**						
**F**	**R**	**Amplicon**	**Ta (˚C)**	**Extn. time (min:sec)**	**Size (bp)**	**Gel Image**
ATTTAGACA CATAGGATC GT	TGTGTATTCAGT CTTTGACA	*aip56* (scaffold AEGA01003780)	50	30 s	871	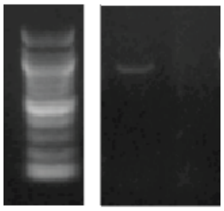
TCAATGAAC TATATTGTTC G	TGCGATTTTATT AATGATAT	*aip56* linking to nearest eukaryotic gene (Mdes005443)	50	50 s	1361	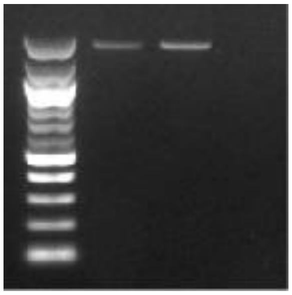
TCCTAATATT AAATTTGCT G	ATGTTAACCTCC ACAGATAT	*lysozyme 1* (Mdes015794)	51	30 s	947	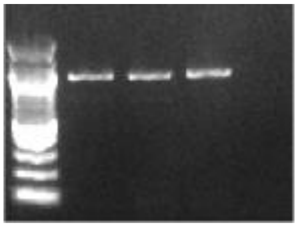
TGAAATGAA TGGTAAGTA CA	TTGAATTATTCA GAAAAATG	*lysozyme 2* (Mdes014481)	51	30 s	815	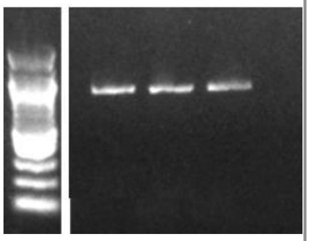

AATCCAAATCATTTAGAAGT	TAGTTTCGATGATTTATTGA	*lysozyme 3* (on scaffold AEGA01003780)	51	30 s	862	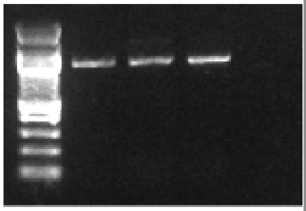
GTCAATATGCACGACTAATA	CAGACAAATCGTACACAGTA	*rhs*	51	30 s	1100	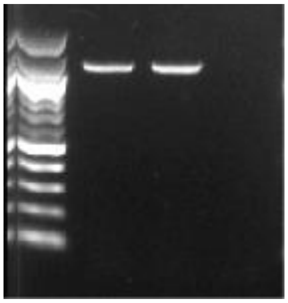
TACTGTGTACGATTTGTCTG	AAATCTTGACTGATACCATC	*rhs*	51	50 s	1541	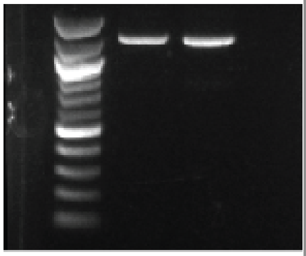
GAATATCAAGATTGACCAGT	TTCACTTTCTGAAGACTCTC	*rhs* linked to the nearest eukaryotic gene (Mdes011033)	51	50 s	1626	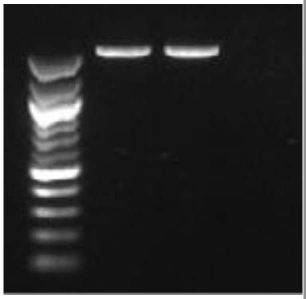

Table S7. Accession numbers for marker genes included on Cecidomyiidae species tree.

**Species**	**Genome**	**Transcriptome**	**CO1 accession #**	**CAD accession #**	**EF1a accession #**	**28S accession #**
*Bibio marci**	NA	SRX314826	KT316846.1	KX453730.1	NA	KJ136761.1
Ca. subobsoleta	GCA_011634745.1	NA	KT316873.1	KX453747.1	MG684878.1	KP288821.1
Co. nasturtii	GCA_009176525.2	SRX6853821	EU812560.1	XM_031771980.1	XM_031764621.1	NA
M. destructor	GCA_000149185.1	SRX516926	EU375697.1	FJ040625.1	AF085227.1	FJ040514.1
Si. mosellana	GCA_009176505.1	SRX252524	KC769209.1	MN191486.1	VUAH01006101.1	MN201531.1

*Outgroup.

Table S8. Details of phylogeny construction for the five identified horizontally transferred genes.

Gene	Queries	# Tips	# Sites	Model	LogL	BIC
AIP56	*Drosophila bipectinata* (XP_017099943.1, AIP56 domain)<br>*Co. nasturtii* (XP_031636937.1, XP_031641113.1)<br>*M. destructor* (this manuscript)<br>*Si. mosellana* (this manuscript<br>APSE1 (NP_050970.1)	90	575	LG+G4	-40095.6183	81322.314
CdtB	Candidatus *Hamiltonella defensa* (XP_016857353.1)<br>*Co. nasturtii* (XP_031641203)<br>*D. ananassae* (XP_014760894.1)<br>*D. biarmipes* (XP_016950904.1)<br>*Myzus persicae* (XP_022165116.1)<br>*Scaptomyza flava* (QDF82162.1).	76	367	LG+F+I+G4	-27554.2607	56112.433
Lysozyme	APSE-2 (YP_002308525.1)<br>*Co. nasturtii* (XP_031638744.1)<br>*M. destructor* (this manuscript).	172	154	WAG+I+G4	-43487.1457	90213.052
SltxB	APSE1 (NP_050968.1)<br>*C.nasturtii* (XP_031619577.1, XP_031619578)<br>*Burkholderia ambifaria* (WP_175804727.1)<br>*Si. mosellana* (copies identified in this manuscript).	23	117	cpREV+G4	-3435.2904	7089.3504
RHS	Bacteriophage APSE3 (CAB3775397.1)<br>*Candidatus* Hamiltonella defensa (ATW32053.1)<br>*Si. mosellana* (copies identified in this manuscript)<br>*M. destructor* (Mdes011034)	188	648	WAG+F+I+G4	-312544.08	628028.7246

Table S9. Evaluation of possible contamination of non-cecidomyiid eukaryotic HGTs.

Species	Gene	Protein ID	Scaffold	Scaffold Coordinates	Scaffold Size	Notes	Assessment
Operophtera brumata	AIP56	KOB51764.1	JTDY01021334.1	OBRU01_27280	1637	No other eukaryotic genes encoded on this scaffold.	No support.
Operophtera brumata	AIP56	KOB68849.1	JTDY01003904.1	OBRU01_17759	48412	Encoded with several other eukaryotic genes, such as alpha-tubulin (KOB68848.1).	Support.
Operophtera brumata	AIP56	KOB69574.1	JTDY01003427.1	OBRU01_16649	53923	Encoded with several other eukaryotic genes, such as an olfactory receptor (KOB69575.1).	Support.
Danaus plexippus plexippus	AIP56	OWR45007.1	AGBW02012468.1	KGM_202948	4568	No other eukaryotic genes encoded on this scaffold.	No support.
Thrips palmi	AIP56	XP_034247031.1	NW_023144630.1	XM_034391140.1	13508960	Predicted to have introns. Expressed in several life stages (BioProject PRJNA498538). Encoded with several other eukaryotic genes, such as stearoyl CoA desaturase (XP_034247036.1).	Support.
Bactrocera latifrons	AIP56	XP_018782911.1	NW_017534797.1	XM_018927366.1	942341	Encoded with several other eukaryotic genes, also syntenic within the three species.	Very strong support.
Bactrocera dorsalis	AIP56	XP_019847121.1	NW_011876381.1	XM_019991562.1	2837044	Encoded with several other eukaryotic genes, also syntenic within the three species. The two are in tandem in B. dorsalis.	Very strong support.
Bactrocera oleae	AIP56	XP_014102626	NW_021667836.1	XM_014247151.2	6418216	Encoded with several other eukaryotic genes, also syntenic within the three species.	Very strong support.
Bactrocera dorsalis	AIP56	XP_029407604.1	NW_011876381.1	XM_029551744.1	2837044	Encoded with several other eukaryotic genes, also syntenic within the three species. The two are in tandem in B. dorsalis.	Very strong support.

Frankliniella occidentalis	AIP56	XP_026288677.1	NW_020292711.1	LOC113213739	982128	Predicted to have introns. Expressed in several life stages (BioProject PRJNA454326). Encoded with several other eukaryotic genes, such as protein obstructor-E (XP_026288642.1).	Support.
Cinara cedri	AIP56	VVC38548.1	CABPRJ010001480.1	CABPRJ010001480.1:48727..49600, 54050..54180	303121	Encoded with several other eukaryotic genes, such as Zinc finger (VVC38550.1).	Support.
Bradysia coprophila	AIP56	XP_037050353.1	NW_023503972.1	LOC119084473	6689181	Encoded with several other eukaryotic genes, such as eukaryotic translation initiation factor 6 (XM_037194457.1). Expressed in several life stages (PRJNA291918). Syntenic with *aip56* in another Bradysia odoriphaga (KAG4066459.1).	Very strong support.
Acyrthosiphon pisum	AIP56	NP_001313599.1	NC_042496.1	LOC100569341	42333646	Encoded with several other eukaryotic genes, such as PSME3-interacting protein (XM_001950757.4). Expressed in several lifestages (PRJNA277874).	Support.
Aphis gossypii	CdtB	XP_027840804.1	NW_021006093.1	LOC114122360	32855	Encoded on a scaffold with bacterial genes, such as XP_027850838 (ATP-binding protein). Strong likelihood this is contamination.	Contaminant.
Dermatophagoides pteronyssimus	Lysozyme	XP_027195130.1	NW_020873402.1	LOC113789752	2731825	Predicted to have intron (XM_027339329.1). Encoded with several other eukaryotic genes, such as mRNA galectin-8-like (XM_027339328.1).	Support.
Bradysia coprophila	Lysozyme	XP_037029278.1	NW_023503313.1	LOC119069325	5404297	Encoded with several other eukaryotic genes, such as Ser/Thr protein kinase (XM_037173386.1). Predicted to have introns. Expressed in several life stages (PRJNA29918), particularly embryos.	Support.

Table S10. Specifics of forced monophyletic trees used in the Approximately Unbiased (AU) topological tests. Asterisks indicate that the only sampled insects are cecids, so an additional forced phylogeny is redundant.

**AIP56**	
Cecid-Mono	• VUAH01006225.1-11973222-11973743 *(Sitodiplosis mosellana)*<br>• VUAH01001532-2410-2829 *(Sitodiplosis mosellana)*<br>• GL501532-1200694-1201296 *(Mayetiola destructor)*<br>• XP_031636937.1 (*Contarinia nasturtii)*<br>• XP_031641113.1 *(Contarinia nasturtii)*
Cecid/APSE-Mono	• Cecid-Mono +<br>• ACJ10079.1 (APSE-5)<br>• CAB4327916.1 (APSE-7)<br>• WP_015874047.1 (*H. defensa*)<br>• WP_015979995.1 (*H. defensa*)
Insects-Mono	• Cecid-Mono +<br>• KOB51764.1 (*Operophtera brumata)*<br>• KOB68849 .1 (*Operophtera brumata*)<br>• KOB69574.1 (*Operophtera brumata)*<br>• OWR45007.1 (*Danaus plexippus*)<br>• XP 018782911.1 (*Bactrocera latifrons*)<br>• XP 019847121.1 (*Bactrocera dorsalis*)<br>• XP 014102626.1 (*Bactrocera oleae*)<br>• XP 029407604.1 (*Bactrocera dorsalis*)<br>• XP 026288677.1 (*Franklinealla occidentalis)*<br>• NP 001313599.1 (*Acyrthosiphon pisum*)<br>• VVC38548.1 (*Cinara cedri*)<br>• XP 037050353.1 (*Bradysia coprophila*)<br>• XP 034247031.1 (*Thrips palmi*)
**CdtB**	
Cecid-Mono	• XP._031639861.1 (*Contarinia nasturtii)*<br>• XP_031641203.1 (*Contarinia nasturtii)*
Cecid/APSE-Mono	• Cecid-Mono +<br>• WP_016857353.1 (*H. defensa)*<br>• WP_100096556.1 (*H. defensa*)<br>• WP_171967391.1 (*H. defensa)*<br>• CAB3623624.1 (APSE-7)<br>• CAB3623637.1 (APSE-7)<br>• CAB3775433.1 (APSE-7)<br>• CAB3775476.1 (APSE-7)
Insects-Mono	• Cecid-Mono +<br>• XP_0147608941 (*Drosophila ananassae)*<br>• XP_0170999431 *(Drosophila bipectinata)*<br>• XP_0170999701 (*Drosophila bipectinata)*<br>• XP_0169509041 (*Drosophila biarmipes)*<br>• XP_0221631161 (*Myzus persicae)*<br>• M-cerasi (from Verster et al 2019) - (*Myzus cerasi)*<br>• QDF821591 (*Scaptomyza pallida)*<br>• QDF821601 (*Scaptomyza nr. flava)*<br>• QDF821621 (*Scaptomyza flava)*<br>• QDF821631 (*Drosophila primaeva)*

**Lysozyme**	
Cecid-Mono	• AEGA01024319 3788-4225 (*Mayetiola destructor)*<br>• AEGA01007297 120-560 (*Mayetiola destructor)*<br>• GL501497 230410-230853 (*Mayetiola destructor)*<br>• NW02201606 2607199-2606738 (*Contarinia nasturtii)*<br>• XP_031638744.1 (*Contarinia nasturtii)*<br>• NW02201606 2605394-2605837 (*Contarinia nasturtii)*
Insects-Mono	• Cecid-Mono +<br>• XP_027195130 (*Dermatophagoides pteronyssus)*<br>• XP_037029278.1 (*Bradysia coprophila)*
Cecid/APSE-Mono	• Cecid-Mono +<br>• ACJ10096.1 (APSE)<br>• YP_002308525.1 (APSE-2)<br>• WP_171967290 *(Hamiltonella defensa)*<br>• WP_171967609 (*Hamiltonella defensa)*<br>• WP_174889564 (*Hamiltonella defensa)*
**RHS**	
Cecid-Mono	• Mdes011034 (*Mayetiola destructor)*<br>• VUAH01005136 999-6117 (*Sitodiplosis mosellana)*
Insect-Mono	*
Cecid/APSE-Mono	• Cecid-Mono +<br>• ACJ10121.1 (APSE-3)<br>• ATW32053.1 (*Hamiltonella defensa)*<br>• AMD43718.1 (APSE)<br>• CAB3775397.1 (APSE-3)
**SltxB**	
Cecid-Mono	• VUAH1000016.1 899256-899522 (*Sitodiplosis mosellana)*<br>• VUAH0100016.1 901146-901313 (*Sitodiplosis mosellana)*<br>• VUAH01000060.1 423461-423673 (*Sitodiplosis mosellana*)<br>• VUAH01000016.1 896355-896597 (*Sitodiplosis mosellana*)<br>• VUAH01000060.1 425037-425252 (*Sitodiplosis mosellana*)<br>• VUAH01000016.1 900260-900502 (*Sitodiplosis mosellana*)<br>• VUAH01006166.1 4792842-4792842 (*Sitodiplosis mosellana*)<br>• XP_031619577.1 (*Contarinia nasturtii)*<br>• XP 031619578.1 (*Contarinia nasturtii)*
Insect-Mono	*
Cecid/APSE-Mono	• Cecid-Mono +<br>• ACJ10077.1 (APSE-5)<br>• NP_050968.1 (APSE-1)
Cecid/Proteobacteria	• Cecid-Mono +<br>• ALI57234.1 (*Escherichia coli)*<br>• EES5291716.1 (*Escherichia coli)*<br>• EFI6751557.1 (*Escherichia coli)*<br>• WP_069985170.1 (*Escherichia coli)*<br>• WP_101973811.1 (*Escherichia coli)*<br>• WP_141044085.1 (*Escherichia coli)*<br>• WP_095907600.1 (*Enterobacter cloacae)*

Table S11. Accession IDs for protein sequences used in structural prediction alignments.

Taxon	CdtB	Lysozyme	RHS	SltxB
**Metazoa**				
Co. nasturtii	XP_031641203.1, XP_031639861.1	XP_031638744, NW2201606 [2605394,2605837], NW02201606 [2607199,2606738]	–	XP_031619577.1, XP_031619578.1
M. destructor		AEG01007297 [120-560], AEG01024319 [3788,4225], GL501497 [230410,230853]	GL501425[246422,251816]	
Si. mosellana			VUAH01006948[8,4950]	UAH1000016.1 [900260,900502], [896355,896597], VUAH01000060.1 [425037,425252], [423461,423673], VUAH01000016.1 [899256,899522]
D. ananassae	XP_014760894.1			
D. biarmipes	XP_016950904.1			
Scaptomyza flava	QDH44045.1			
Myzus persicae	XP_022163116.1			
**Fungi**				
Fusarium decemcellulare		KAF5009760.1)		
Coprinopsis cinerea		XP_001840847.1		
**Prokaryote**				
Enterobacteriophage T4		ENLYS_BPT4		
Enterobacteriaphage H19				STXB_BPH19

Staphylococcus phage SA1		D2K0A1		
Bacteriophage APSE1				NP_050968.1
Bacteriophage APSE2	AGX01517.1	YP_002308525.1		
Bacteriophage APSE3			CAB3775397.1	
Bacteriophage APSE5		ACJ10082.1		ACJ10077.1
Escherichia coli	Q46669.1		AAB18570.1	EF|6751557.1
Poplar bacterium				RYX79668.1
Haemophilus ducreyi	AKO43951.1			
Vibrio cholerae			Q9KS45.1	
Salmonella enterica			ECF7068452.1	
Bacillus subtilis			WP_192858111.1	
Yersinia entomophaga			ABG33864.1	
Xenorhabdus vietnamensis			WP_086110724.1	
**Outgroups**				
Bovine DNAse I	P00639.3			

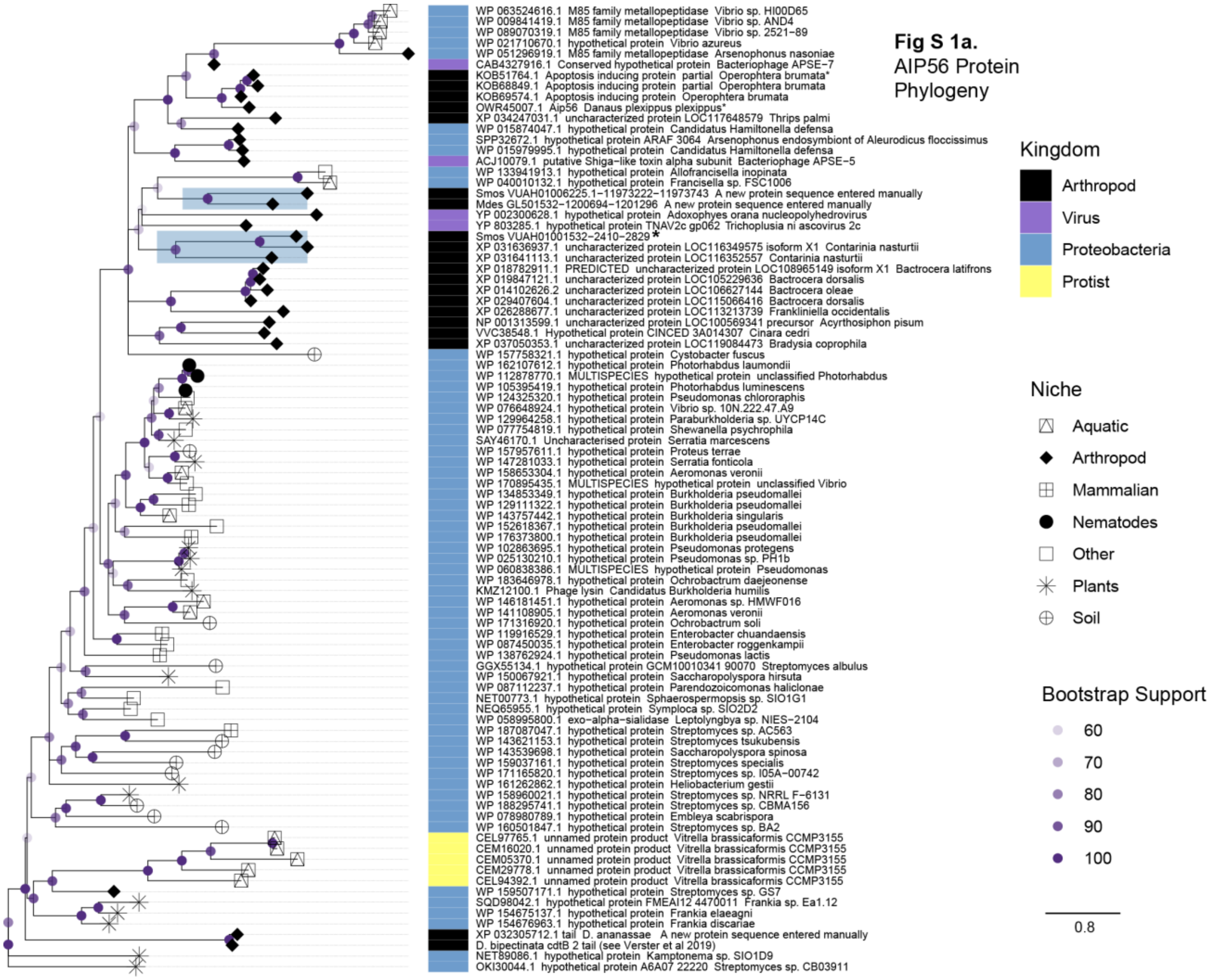

Fig S 1a. AIP56 Protein Phylogeny

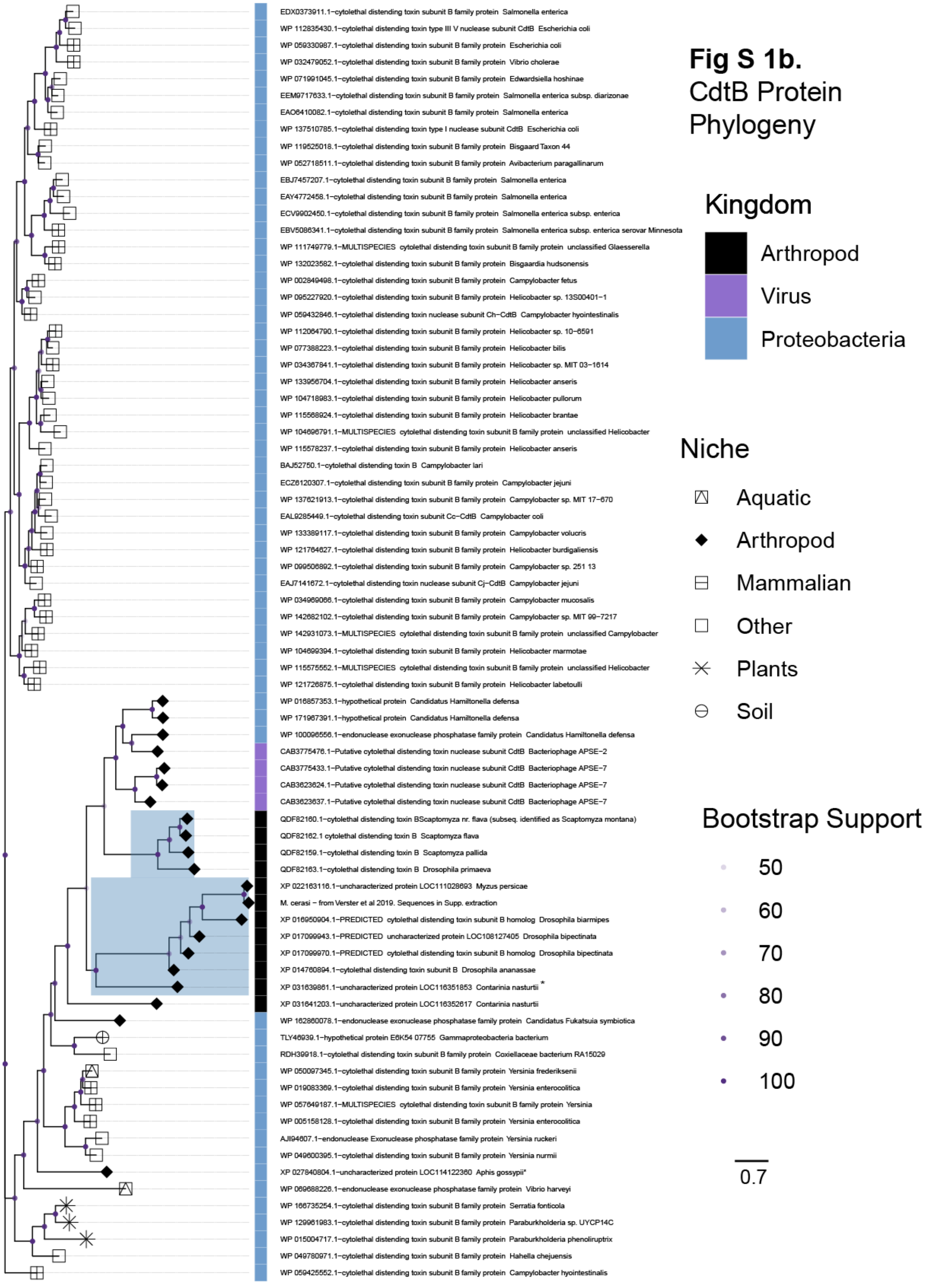

Fig S 1b. CdtB Protein Phylogeny

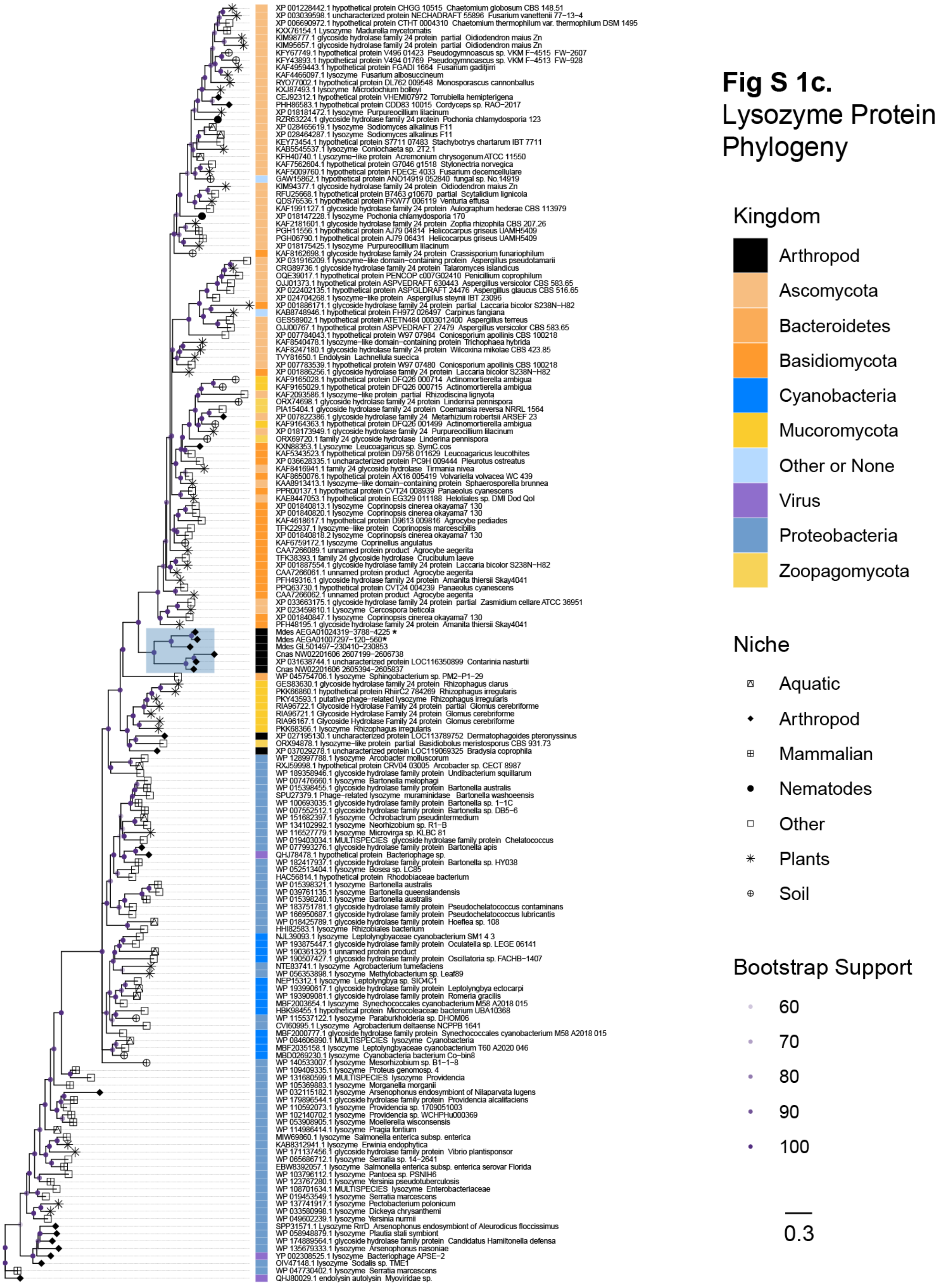

Fig S 1c. Lysozyme Protein Phylogeny

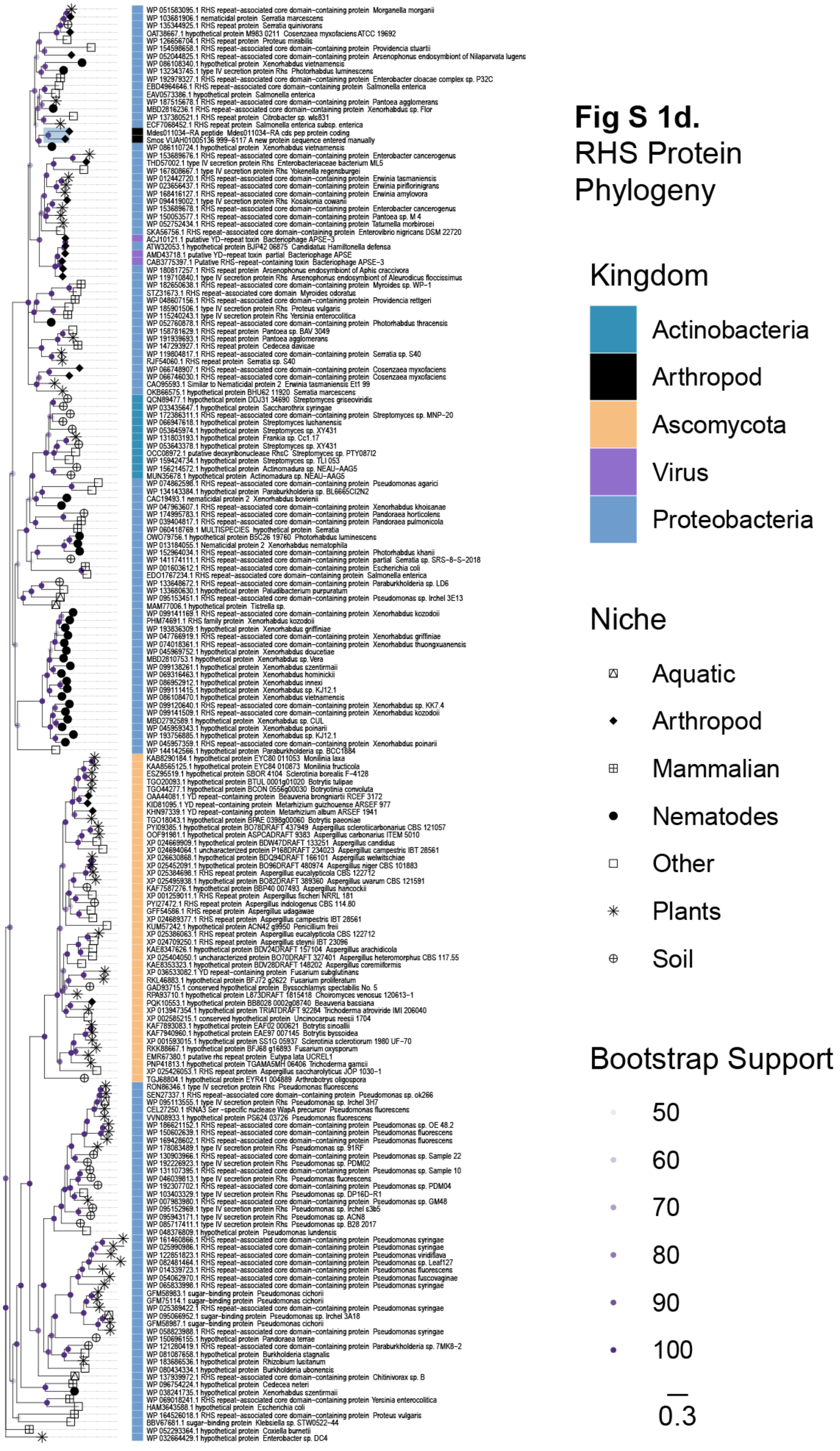

Fig S 1d. RHS Protein Phylogeny

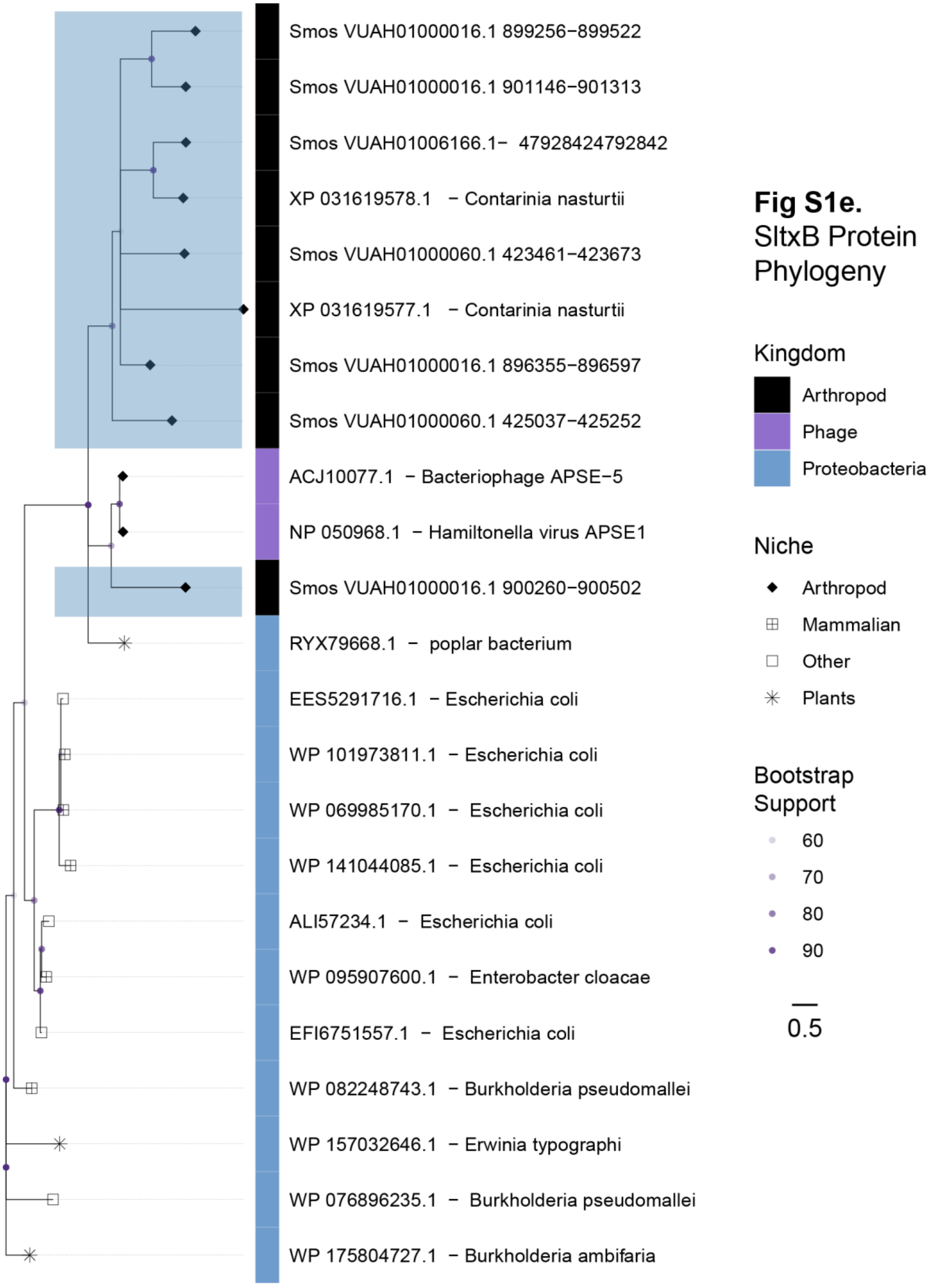

Fig S1e. SltxB Protein Phylogeny

Fig S1. Complete Maximum Likelihood protein phylogenies for five genes transferred to the Cecidomyiidae lineage reveal possible donor provenance. Phylogenies show taxonomic and niche information for each tip. HTG clades discussed in the manuscript are highlighted in blue. Bootstrap values (*n*=1000) are depicted by the opacity of nodes. Asterisks indicate insect taxa that may be contaminants (see **Supplementary File 1** and **Table S9**). Scale bars indicate substitutions per site. **Figs S1a-e** show, respectively: AIP56, CdtB, Lysozyme, RHS, and SltxB.

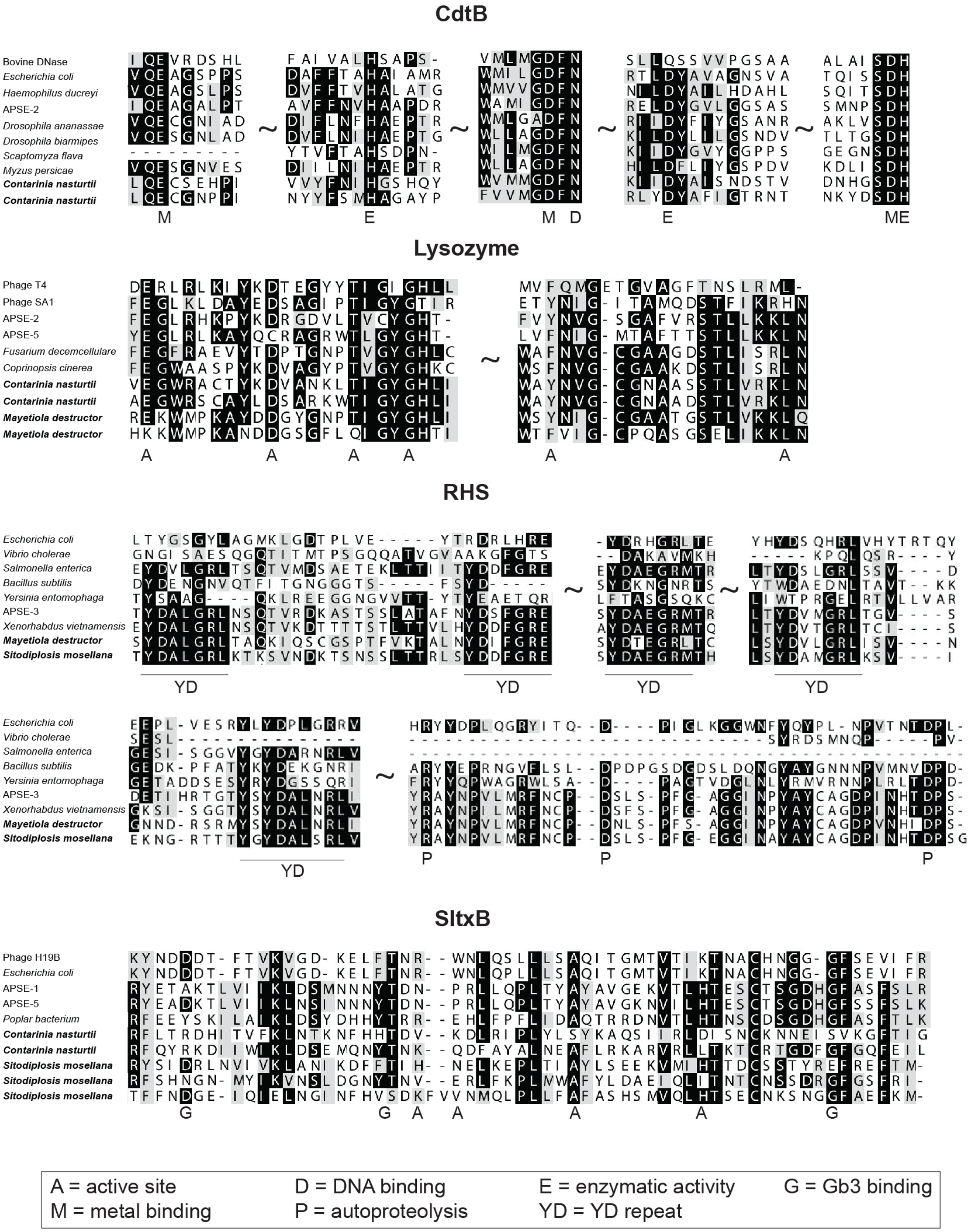

Fig S2. Protein alignments of toxin proteins transferred to gall midges reveal that many critical residues are conserved (see **Table S11** for accession IDs of representative sequences).

FIG S3a.
AIP56, HGT-only phylogeny.

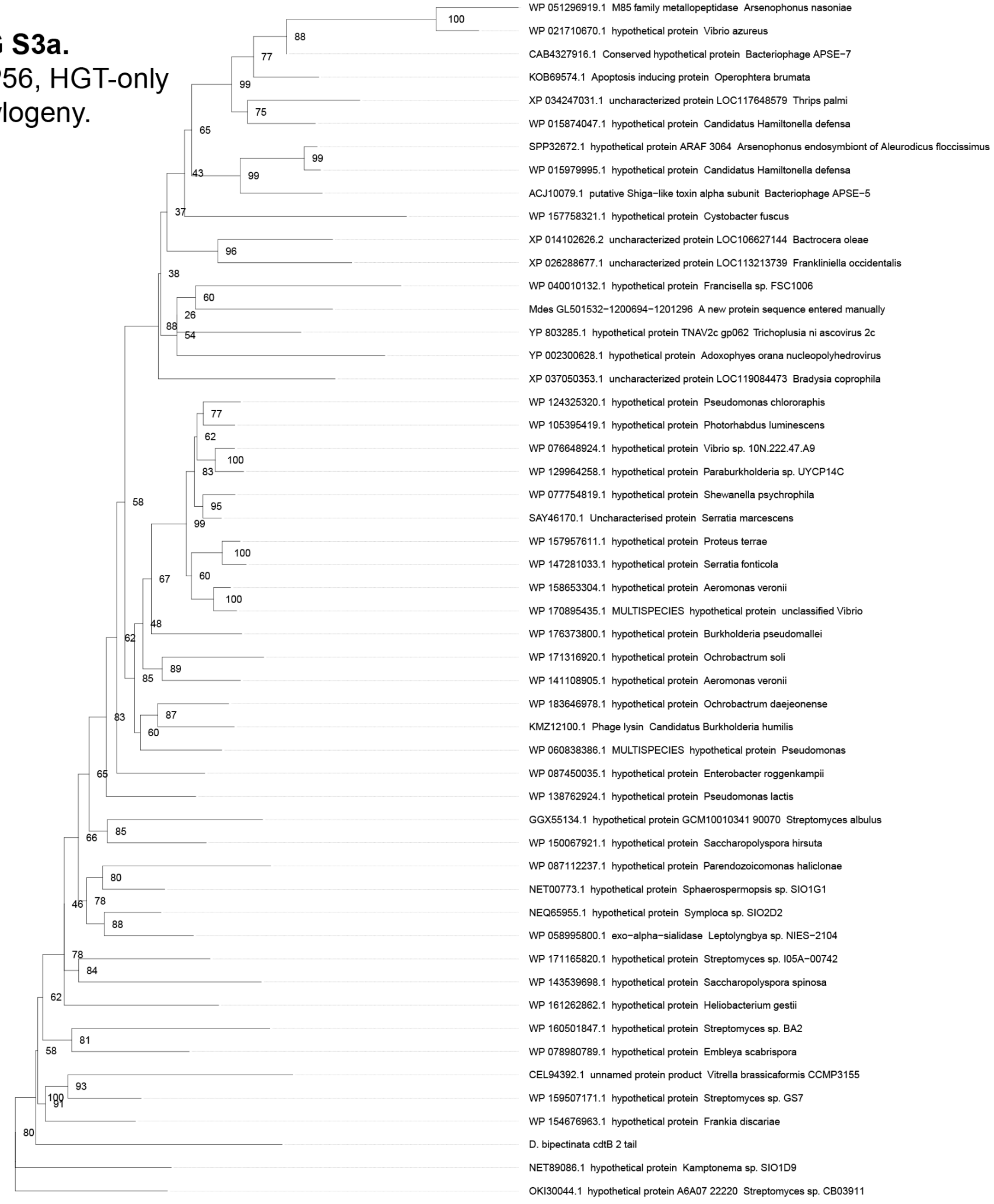

FIG S3b.
CdtB, HGT-only phylogeny.

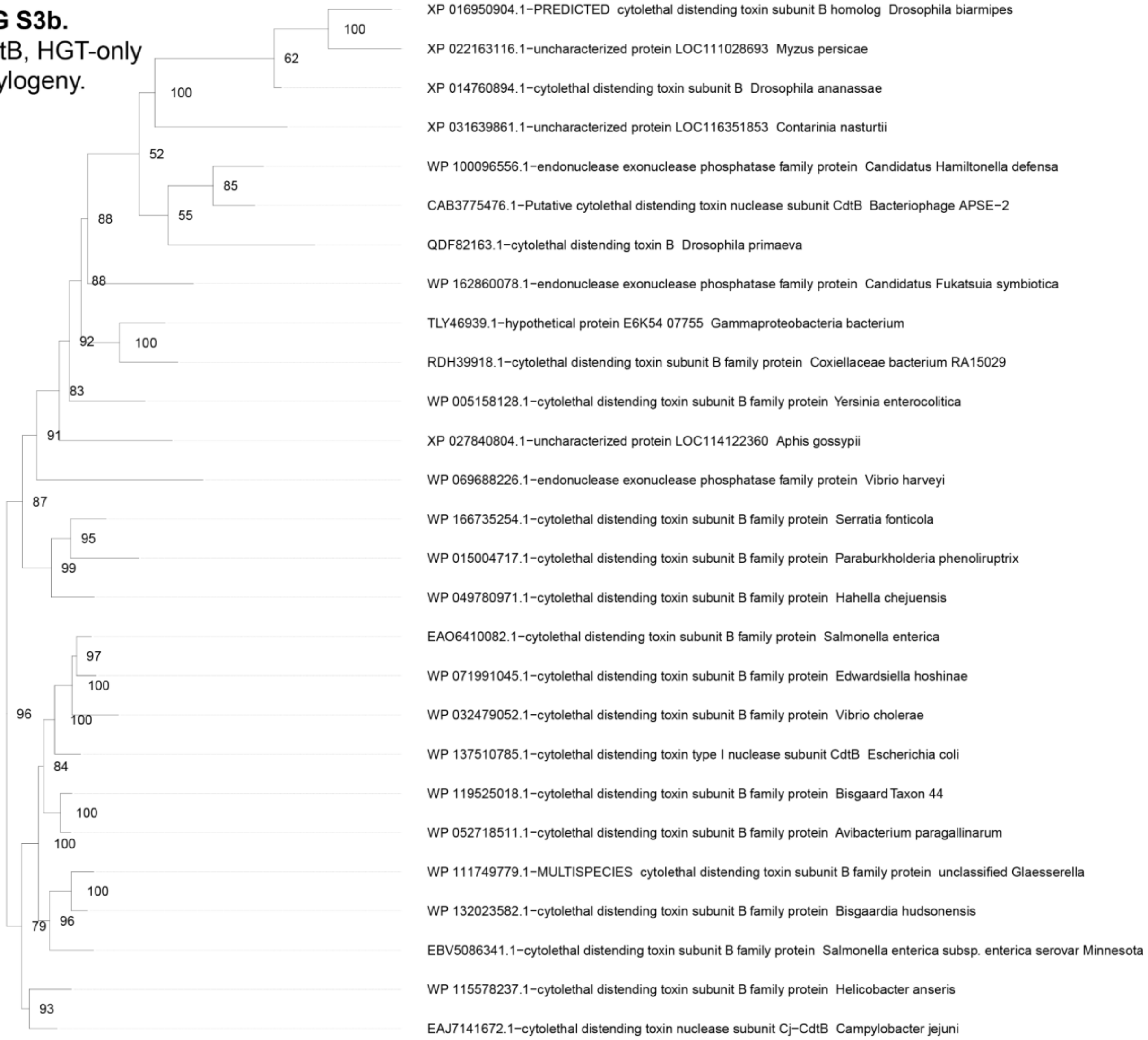

FIG S3c. Lysozyme, HGT-only phylogeny.

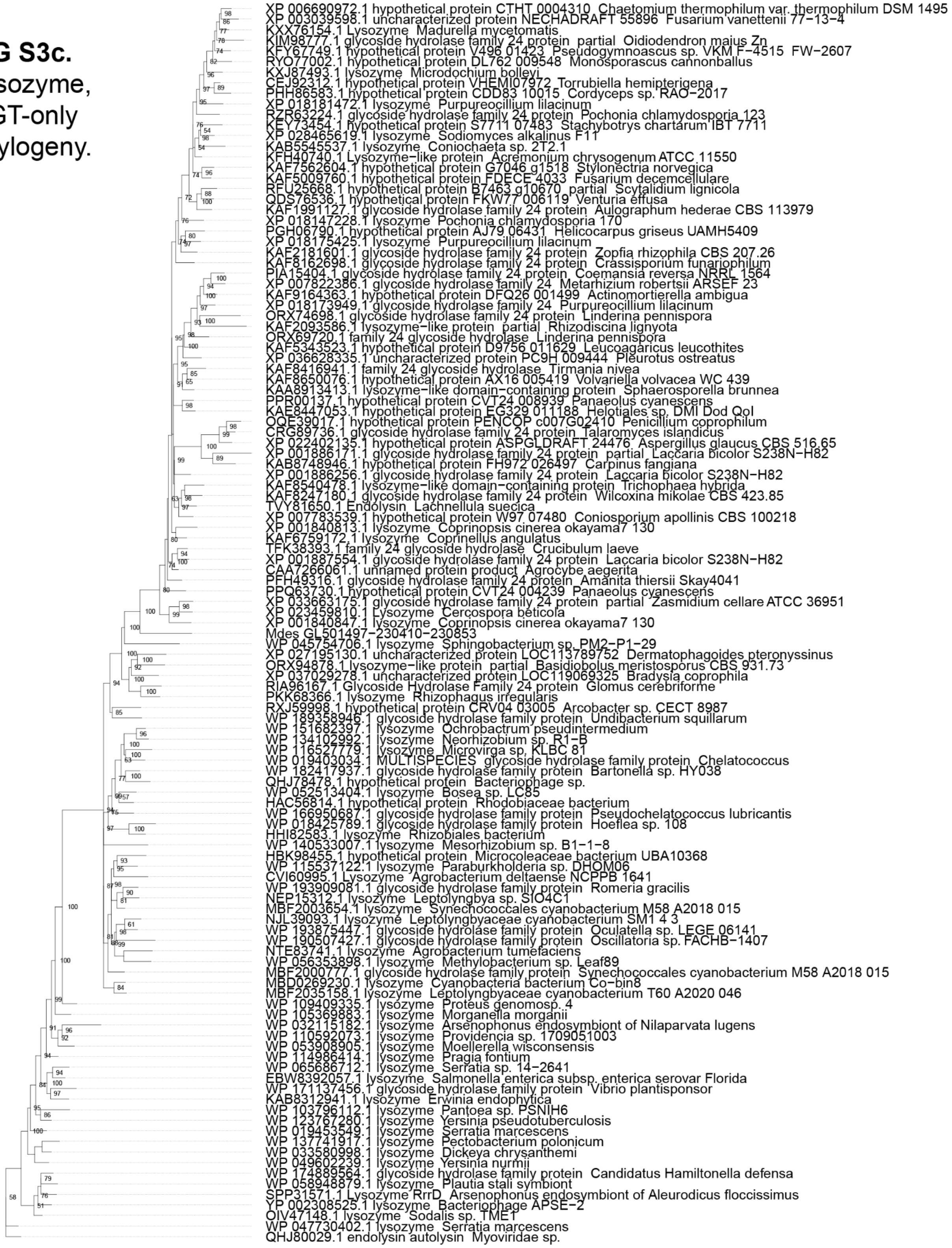

FIG S3d. RHS, HGT-only phylogeny.

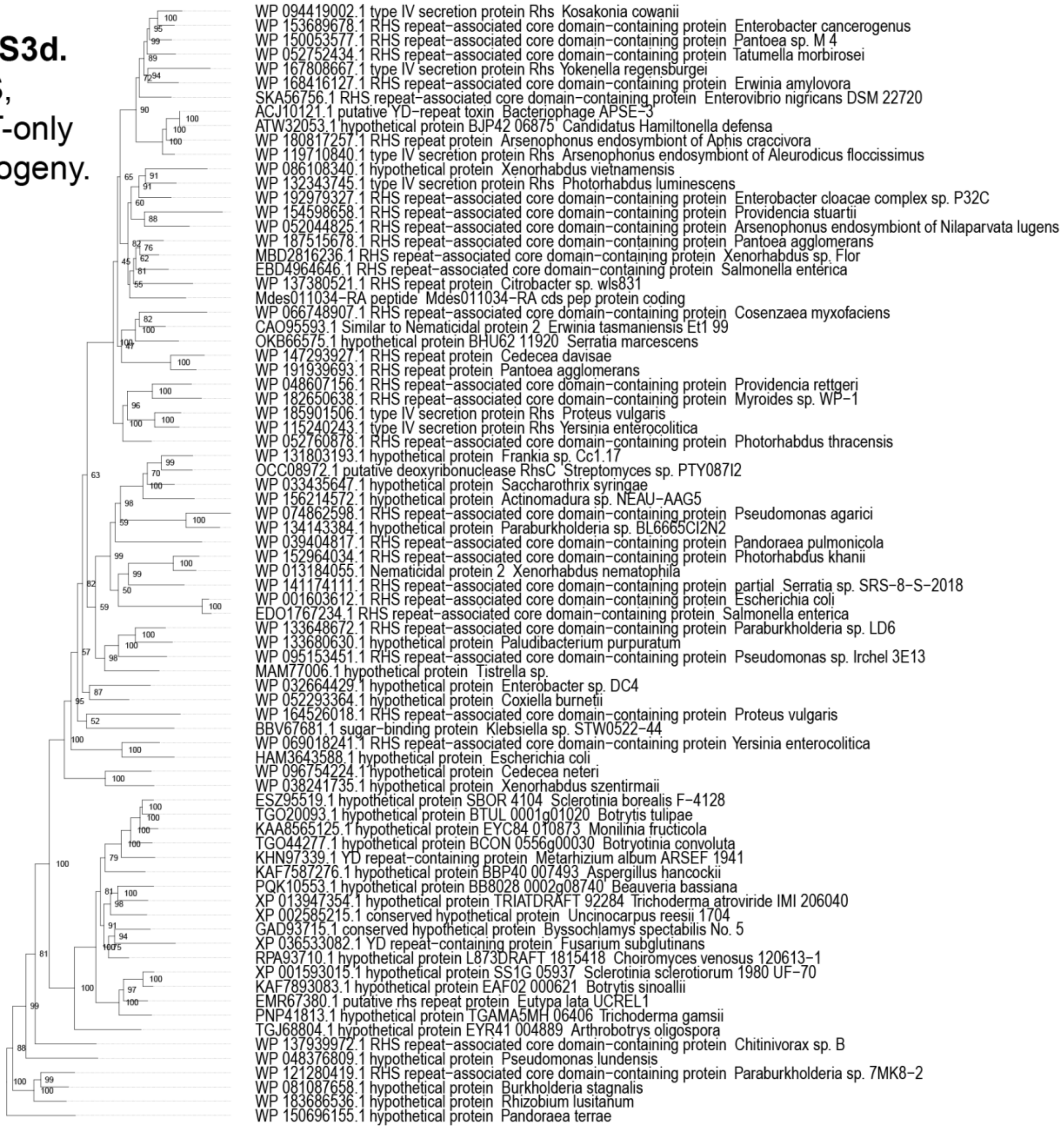

FIG S3e. SltxB, HGT-only phylogeny.

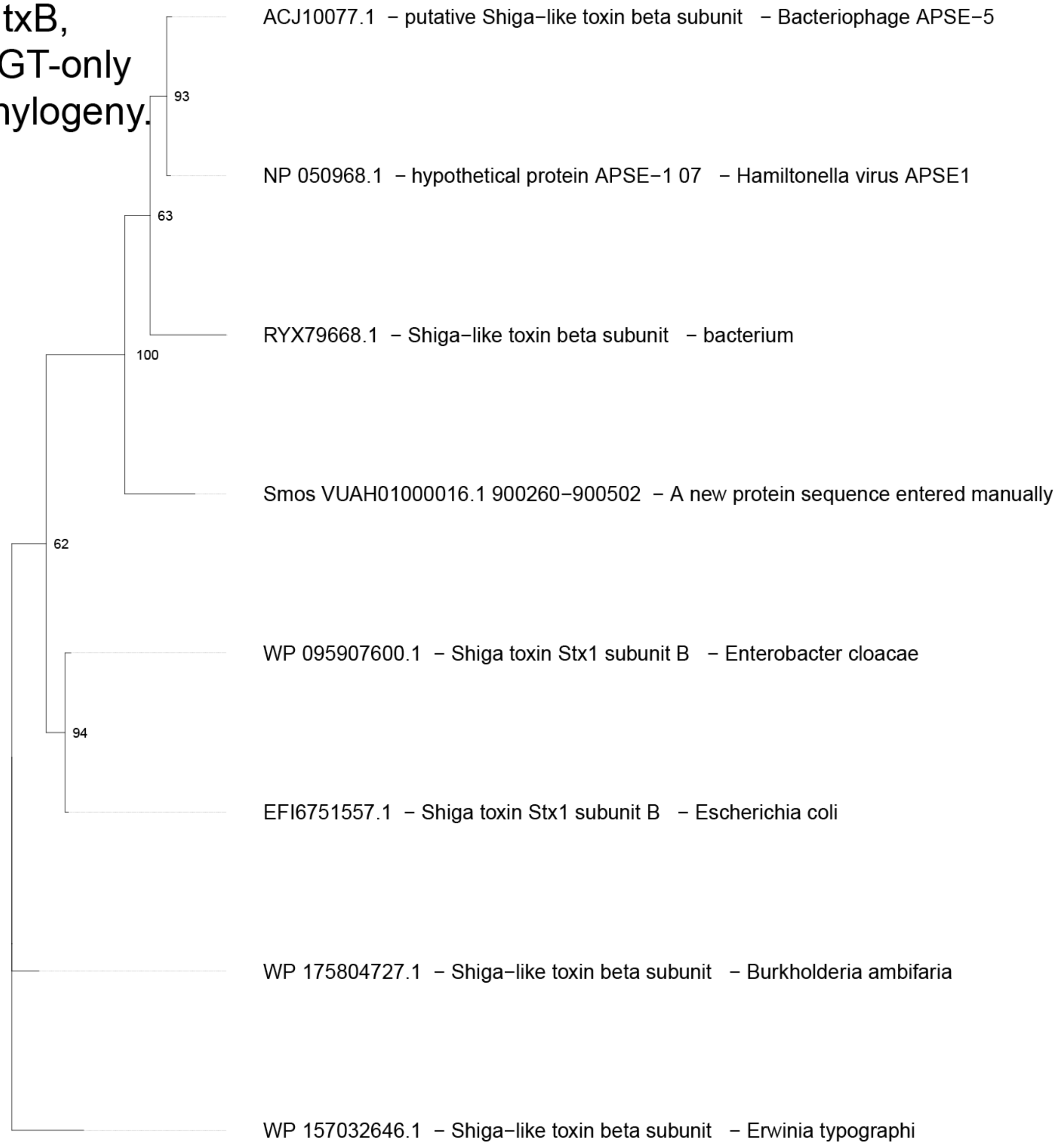

Fig S3f.

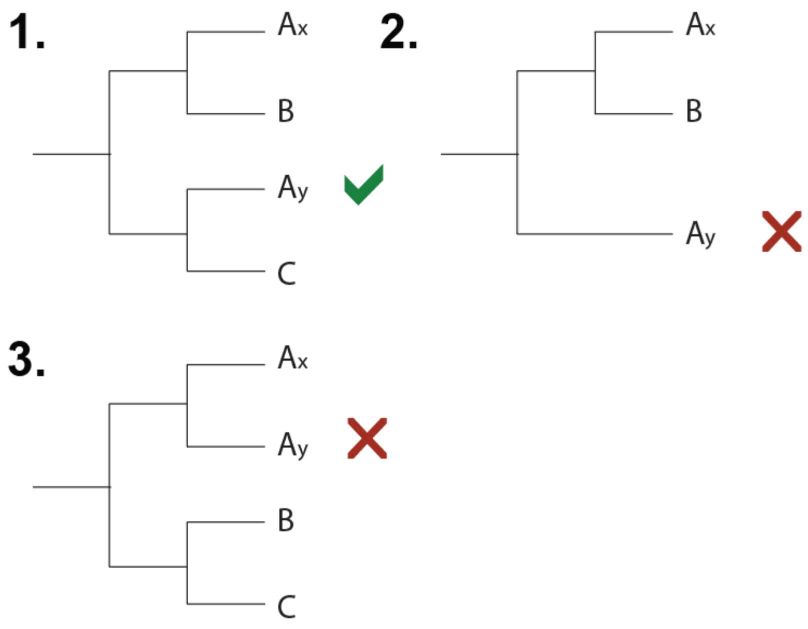

Fig S3. HGT-only protein phylogenies phylogenies. Phylogenies have been trimmed to only include tips that were likely passed down via HGT. Asterisks indicate insect clades that may be contaminants (see **Table S9**). **Figs S3a-e** show, respectively: AIP56, CdtB, Lysozyme, RHS, and SltxB. **Fig S3f** shows what taxa (indicated by letters on the phylogeny) were retained (green ✓), and which were removed (red X), in constructing the HGT-only phylogenies.

Supplementary File 1 Legend. These analyses support the finding that the horizontally transferred genes identified in this study are not due to microbial contamination. 'Species' column shows the species in which the HGT occurred. 'APSE' and 'Protein ID' columns show the APSE strain and GenBank IDs of query sequences identified in the cecidomyiid genomes. For the 'Protein Name' column, we report a summary of the BLASTP results if they appear to correspond to one or more characterized proteins. In the 'E-value' column, we report the lowest E-value in the case of multiple APSE protein queries. In some cases, a single TBLASTN query resulted in hits to multiple genomic 'ranges' on the same scaffold. If the subject sequences shared high AA identity (>90%) throughout multiple ranges, we considered these to be evidence of duplications of the HTG, and the E-values for each individual 'range' was reported in separate rows. 'Scaffold' and 'Scaffold Size' coordinates reflect GenBank accessions and associated lengths unless otherwise noted. 'HTG Coordinates' column reflects the TBLASTN reported ranges, unless the HTG has been annotated, in which case the annotation ID is shown. In the 'Other Eukaryotic Genes' column, we report if we found evidence of *bona fide* eukaryotic genes (Yes/No) on the scaffold. In the 'Intron' and 'Exon Coordinates' column, we indicate the number of introns predicted by either annotations specific to the species or Augustus annotations. In some cases, Augustus did not predict any genes in the region of interest, in which case we reported 'NGP' for 'No Gene Predicted.' Where the HTG does not have an associated annotation ID, we report the Augustus-predicted exon coordinates. For the 'BWA' and 'Transcription' column, we report mean read depth and standard deviation of genomic and transcriptomic reads, respectively. Genes that have a high likelihood of being contaminants (i.e. on scaffolds <10 kb without other *bona fide* eukaryotic genes encoded on the same scaffold) are highlighted light grey and with an asterisk.

Supplementary File 2 Legend. Taxonomic and niche information for species in protein phylogenies shown in **Fig S1**.

Supplementary File 3 Legend. Newick file containing complete phylogenies used in this study. The order of trees is, respectively: AIP56, CdtB, Lysozyme, RHS, and SltxB.

Supplementary Bibliography

1. Blake MC, Jambou RC, Swick AG, Kahn JW, Azizkhan JC. 1990. Transcriptional initiation is controlled by upstream GC-box interactions in a TATAA-less promoter. Mol. Cell. Biol. 10:6632–6641.
2. Cavener DR. 1987. Comparison of the consensus sequence flanking translational start sites in *Drosophila* and vertebrates. Nucleic Acids Res. 15:1353–1361.
3. Elliott B, Olfert O, Hartley S. 2011. Management practices for wheat midge, *Sitodiplosis mosellana* (Géhin). Prairie Soils and Crops. 4:8-13.
4. Graves BJ, Johnson PF, McKnight SL. 1986. Homologous recognition of a promoter domain common to the MSV LTR and the HSV tk gene. Cell. 44:565–576.
5. Katoh K, Standley DM. 2013. MAFFT multiple sequence alignment software version 7: improvements in performance and usability. Mol. Biol. Evol. 30:772–780.
6. Kelley LA, Mezulis S, Yates CM, Wass MN, Sternberg MJE. 2015. The Phyre2 web portal for protein modeling, prediction and analysis. Nat. Protoc. 10:845–858.
7. Kutach AK, Kadonaga JT. 2000. The downstream promoter element DPE appears to be as widely used as the TATA box in *Drosophila* core promoters. Mol. Cell. Biol. 20:4754–4764.
8. Proudfoot NJ. 2011. Ending the message: poly(A) signals then and now. Genes Dev. 25:1770–1782.
9. Raymondjean M, Cereghini S, Yaniv M. 1988. Several distinct ‘CCAAT’ box binding proteins coexist in eukaryotic cells. Proc. Natl. Acad. Sci. U. S. A. 85:757–761.
10. Shine J, Dalgarno L. 1974. The 3’-terminal sequence of *Escherichia coli* 16S ribosomal RNA: complementarity to nonsense triplets and ribosome binding sites. Proc. Natl. Acad. Sci. U. S. A. 71:1342–1346.
11. Thomas MC, Chiang C-M. 2006. The general transcription machinery and general cofactors. Crit. Rev. Biochem. Mol. Biol. 41:105–178.
12. Verster KI et al. 2019. Horizontal transfer of bacterial cytolethal distending toxin B genes to insects. Mol. Biol. Evol. 36:2105–2110.
